# Topology Augmented with Geometry in the Assembly of Structural Databases: Kagome Intermetallics

**DOI:** 10.1002/advs.202417041

**Published:** 2025-07-04

**Authors:** Nataliya L. Gulay, Dongsheng Wen, Joshua E. Griffiths, Judith Clymo, Luke M. Daniels, Jonathan Alaria, Matthew S. Dyer, John B. Claridge, Matthew J. Rosseinsky

**Affiliations:** ^1^ Department of Chemistry Materials Innovation Factory University of Liverpool 51 Oxford Street Liverpool L7 3NY UK; ^2^ Department of Computer Science University of Liverpool Ashton Street Liverpool L69 3BX UK; ^3^ Department of Physics The Oliver Lodge Laboratory University of Liverpool Liverpool L69 7ZE UK; ^4^ Leverhulme Research Centre for Functional Materials Design Materials Innovation Factory University of Liverpool 51 Oxford Street Liverpool L7 3NY UK

**Keywords:** database, intermetallic phases, kagome, machine learning, structural nets

## Abstract

Creation of well‐curated databases tailored to specific structural motifs can underpin and drive materials discovery, as the properties of materials are governed by composition and structure. The role of such motifs in directing the intricate interplay between structure and properties is exemplified by intermetallic compounds with structures that contain kagome layers that exhibit a variety of exotic physical states. Two prevailing approaches have previously been applied to identify such materials: evaluation of structural topology or geometry assessment, however, both present limitations if deployed individually. We augment topological screening with geometrical filtering to allow versatile control over the identification of kagome layers. Applying this approach with minimal further constraints labels over 9000 kagome‐containing intermetallics which are assigned to four structural classes, revealing connections between symmetry, composition, direct space structure, and flatband electronic structures in reciprocal space. A machine learning model is used to predict new element combinations that favour the formation of kagome layers. Several highly‐ranked phase fields correspond to known kagome‐containing materials that were absent from the training dataset, demonstrating that the workflow can identify chemistries affording kagome layers. This motivates the extension of the approach beyond kagome to other property‐conferring motifs, such as honeycomb, square planar or triangular plane nets.

## Graphical abstract

The combination of topological filtering with geometrical restraints reliably screens for kagome layers within intermetallic compounds. The resulting comprehensive list of kagome net‐containing intermetallics can be reviewed to identify examples of interest for particular investigations and also used to build ML models to guide the search for further examples.

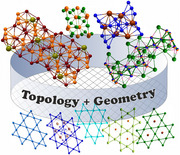

## 1 Introduction

The properties of materials are controlled by the interplay of their structure and composition.^[^ ^1^, ^2^ ^]^ Extensive well‐curated crystallographic databases enable the identification of materials compositions containing specific extended structural motifs that determine critical material properties.^[^ ^3^, ^4^, ^5^ ^]^ Accurate identification of materials containing such motifs will strengthen our ability to connect chemical composition to those structural elements that control properties and also identify materials whose performance could be improved by further compositional control of their structures. In addition to highlighting known materials whose structures favour outstanding properties, or could be tuned to do so, new candidate compositions where these motifs might be expected can be identified, both by manual evaluation and via construction of machine learning models from labelled data.^[^ ^6^ ^]^ The creation of these comprehensive databases tailored for specific research targets is an essential prerequisite for such data‐driven discoveries.^[^ ^7^, ^8^ ^]^

**Figure 1:**
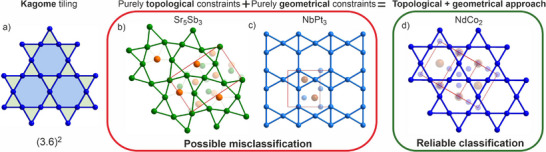
a) Kagome tiling (3.6)^2^ composed of corner‐sharing triangles connected via edges to hexagonal tiles. Examples of materials that could be misclassified as kagome if using: b) a purely topological approach that might disregard chemical bonding (e.g., Sr_5_Sb_3_^[^ ^24^ ^]^ which contains highly distorted layers); c) or a purely geometrical approach that searches for 4 equidistant nearest neighbors with 60°/120° angles (e.g., NbPt_3_^[^ ^25^ ^]^ that does not contain a kagome layer). The combination of both approaches leads to a reliable classification extending to structures with lower symmetries than hexagonal, exemplified by d) low‐temperature NdCo_2_^[^ ^26^ ^]^ with space group *Imma*.

An example of the intricate interplay between structure and properties is the control exerted by kagome layers on the magnetic and electronic properties of materials.^[^ ^9^, ^10^, ^11^, ^12^ ^]^ The kagome layer (**Figure** **1**a) is formed of a triangle‐hexagonal tiling with the corner‐sharing triangles surrounded by hexagons. Its net symbol (3.6)^2^ indicates that there are always two triangles and hexagons which alternate meeting at each edge.^[^ ^13^ ^]^ The kagome layers are observed in several classes of materials including natural minerals^[^ ^14^ ^]^ and synthetic inorganic compounds (intermetallics and semimetals).^[^ ^12^, ^15^ ^]^ Intermetallic kagome magnets such as Mn_3_Sn have attracted a lot of attention for their potential application in low energy spintronic devices.^[^ ^16^ ^]^ In addition, kagome layers in intermetallics can generate a range of exotic phenomena, including correlated electron orders (e.g., in *A*V_3_Sb_5_ where *A* = K, Rb, Cs),^[^ ^17^ ^]^ topological properties (e.g., Mn_3_Sn, FeSn, or *R*V_6_Ge_6_ where *R* is rare‐earth element),^[^ ^16^, ^18^, ^19^ ^]^ and superconductivity (e.g., in CsCr_3_Sb_5_).^[^ ^20^, ^21^ ^]^ These phenomena arise from the geometrical frustration of the layer. For example, localized spins interacting antiferromagnetically are unable to simultaneously satisfy all of these interactions because of the layer geometry,^[^ ^22^ ^]^ and the layer geometry generates a mixture of linear and flat dispersion in the electronic structure that is associated with the functional electronic and magnetic properties.^[^ ^12^ ^]^ The importance of identifying kagome layers within intermetallics was already recognized in *Pearson*’s Handbook of Crystallographic Data for Intermetallic Phases in 1986.^[^ ^23^ ^]^

There are two major approaches that have been used to search for specific structural fragments such as kagome: to focus on layers with a specific structural topology of the net in direct space by evaluating the connectivity between the atoms;^[^ ^27^ ^]^ or to define *geometrically*‐ideal layers.^[^ ^28^ ^]^ The two examples in Figure 1b,c, illustrate the challenges in using *purely topological* or *purely geometrical methods* to search for kagome layers in crystal structures.

*Topology*‐driven structural filtering in direct space treats the atomic sites in the structure as nodes of a graph which could result in loss of information about the actual chemical bonding within the material. Thus, it does not rule out layers that are so severely distorted geometrically that the key chemical and physical characteristics of the kagome tiling are lost, such as the presence of a recognizable hexagon (Figure 1b).

*Geometrical* assessments originate from the strong relationship between electronic and magnetic properties and the bond lengths and angles of the kagome layer.^[^ ^22^ ^]^ Such approaches were used in the majority of recent works that produced lists of kagome compounds.^[^ ^22^, ^28^, ^29^, ^30^, ^31^, ^32^ ^]^ However, many compounds with distorted kagome layers can retain flat bands^[^ ^33^ ^]^ or even exhibit their own unique topological phenomena,^[^ ^34^, ^35^ ^]^ which makes both the identification of distorted kagome layers and the accurate classification of their relationship to the perfect net important. Simple geometrical methods that consider the nearest‐neighbors, bond lengths, and angles may also lead to misclassification, yielding nets that are not kagome at all (Figure 1c).

Our goal is to provide a comprehensive list of intermetallic solids containing kagome layers that presents as much information as possible about the connection between chemistry and structure. In order to capture the chemistries affording the kagome motif, we need to identify the defining topological connectivity while retaining control of the appropriate definitions of geometry that ensure the identified structures are meaningfully kagome in a chemical context. The incorporation of imperfect nets allows the identification of, for example, density wave distortions that could be suppressed by external fields or compositional change.^[^ ^36^ ^]^ Moreover, inclusion of imperfect entries will highlight chemistries leading to kagome connectivity that can be subsequently tuned to minimize or eliminate distortions.^[^ ^37^ ^]^

In this study, we showcase how the combination of topological and geometrical approaches is beneficial for the creation of a comprehensive database by allowing control of the selection process based on chemical understanding. We apply a two‐step assessment protocol to an initial compound dataset assembled without restrictions, e.g., not restricted by space group symmetry or the presence of structural disorder. This maximizes the number of instances considered and retains entries where a kagome layer is present in a structural context of lower symmetry. Retaining disordered examples both ensures that we capture all chemistries that afford the kagome layer and recognizes that the disorder in a solid may occur in a structural component other than the kagome one. We then first filter this unrestricted dataset based on topology with a light geometrical constraint on connectivity to identify kagome layers based on the graphs present in the solid. The second step is to perform a geometrical classification of the materials by assessing kagome connectivity in terms of their bond length and angle relationship to a perfect undistorted kagome layer. By relating all the layers with kagome connectivity to the perfect kagome layer, we maximize the information accessible from the larger dataset. Augmentation of the topological screening with geometrical filters allows the identification of local kagome topology even in cases where the hexagonal symmetry is no longer preserved (Figure 1d). This strategy combines the strengths of both approaches while overcoming the potential limitations of either individual method. This provides an assessment protocol that can be extended beyond kagome layers to other planar and 3D nets of interest for their control of properties.

This workflow generated a database of >9000 intermetallics containing the kagome layer (available as Supporting Information). This extensive list is curated into sub‐categories according to their exact geometry and mapped onto the arising electronic structures, enabling both comprehensive labelling of kagome‐containing flat band and topological materials and the identification of new families of such compounds for future investigation. This illustrates how the list can be reviewed and the method applied to identify examples of interest for particular investigations. The comprehensive identification of kagome‐generating chemistries is also used to build ML models to guide the search for further examples. Such models will facilitate the structure‐targeted synthetic exploration of intermetallic phase fields and illustrates one of the potential uses of the created database.

## 2 Results and Discussion

### 2.1 Filtering Methodology

**Figure 2:**
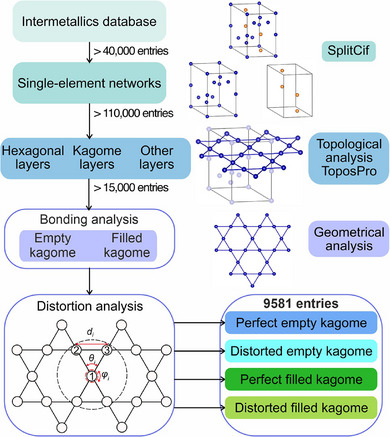
Schematic representation of the classification workflow introduced in this work. For the distortion analysis, θ, *φ*, and *d* represent angles of the triangular tile (close to 60°), angles of the hexagonal tile (close to 120°), and the bond lengths (measured within the triangular tile), respectively. A detailed description of the filtering methods used is given in SI1.

In this study, we start from topological filtering (using the ToposPro software)^[^ ^38^ ^]^ and augment it with further geometrical restraints to identify both perfect and distorted structural fragments. We also employ minimal constraints to the compositions of entries (i.e., focusing on intermetallics but not omitting any disordered or non‐stoichiometric compounds) aiming at obtaining a comprehensive dataset representative of all intermetallic phases in the Inorganic Crystal Structure Database, ICSD.^[^ ^39^ ^]^ The sorting procedure is described by the scheme in **Figure** **2**. It is applied to a database of intermetallic compounds that contain at least two metallic elements (see Figure S1, Supporting Information for the selected elements). The first step uses the custom code SplitCif to generate single‐element files from each structure by creating copies of the initial crystallographic information files with only one elemental species in them. By analysing the sublattices produced by individual elements in this step, it ensures identification of kagome layers that are formed by the same atomic species.

The single‐element entries are processed by ToposPro to evaluate the presence of planar fragments with the kagome topology. One of the few thresholds applied at this stage was the out‐of‐plane distortion that ensured the preservation of planarity. Different thresholds were tested (Table S1; Figure S2, Supporting Information) and the value of 0.1 Å was chosen as it maximized the number of entries while ensuring planarity of the layers. The ToposPro algorithm also shows positive matches for the hexagonal layer, which contains kagome connectivity but is not a kagome layer, see S1.2 and Figure S3 (Supporting Information) for details. Such entries were removed from the list by performing a search specifically for hexagonal layers (see S1.2, Supporting Information). The remaining entries are extracted in the form of graph files and used for further geometrical analysis. For this step, a custom code using the *Pymatgen* package^[^ ^40^ ^]^ was developed to quantify the degree of distortion based on the variations of the bond lengths and angles within triangular and hexagonal tiles, see Figure 2. This process provides an accurate filter for the kagome layers identified through the topological method, while quantifying how the kagome layers are distorted from the perfect geometry.

All kagome layers are classified as empty or filled based on the presence of another atomic species within the hexagonal tiles and with less than 0.15 Å deviation from the plane (“bonding analysis” in Figure 2). We have thus separated the empty and filled layers using geometrical analysis (see Methodology in S1.3, Supporting Information). This step was followed by evaluation of distortions within the kagome layers to distinguish perfect kagome layers from the distorted ones as these classes can possess distinct physical properties. For the analysis, three metrics of the kagome layer have been evaluated: angles close to 60° (θ) of the triangular tile, angles close to 120° (*φ*) of the hexagonal tile, and bond length (*d*), as shown in Figure 2. To distinguish perfect and distorted layers, we defined the normalized standard deviations (*δ*) of three properties of the nets: (1) δ({θ_i_}) for angles ≈ 60°, (2) δ({φ_i_}) for angles ≈ 120°, and (3) δ({*d*}) for the bond lengths measured within the triangular tile. A perfect kagome layer exhibits no distortions in bond lengths and angles; and therefore, δ({θ_i_}), δ({φ_i_}), and δ({d}) will be zero. All entries containing layers with non‐zero deviations are labelled as distorted. For these distorted nets, if the averaged {θ_i_} angles (defined as μ({θ_i_}) in SI Section 1.3) deviate from 60° by >10°, the net is likely severely distorted and is classified as non‐kagome. As a result of this distortion analysis, four classes are obtained for entries that contain perfect‐empty, distorted‐empty, perfect‐filled, and distorted‐filled kagome layers. The detailed description of sorting methods is given in SI1.3.

### 2.2 The Database of Intermetallic Compounds Containing Kagome Layers

The resulting database of intermetallic compounds that contain kagome layers consists of 9581 entries, some of which correspond to the same composition. It represents a more comprehensive database of intermetallic compounds containing kagome layers based on comparison with five recent databases that contain large numbers of entries and a kagome classifier (short summaries are provided in Table S2, Supporting Information).^[^ ^22^, ^28^, ^30^, ^31^, ^41^ ^]^

**Figure 3:**
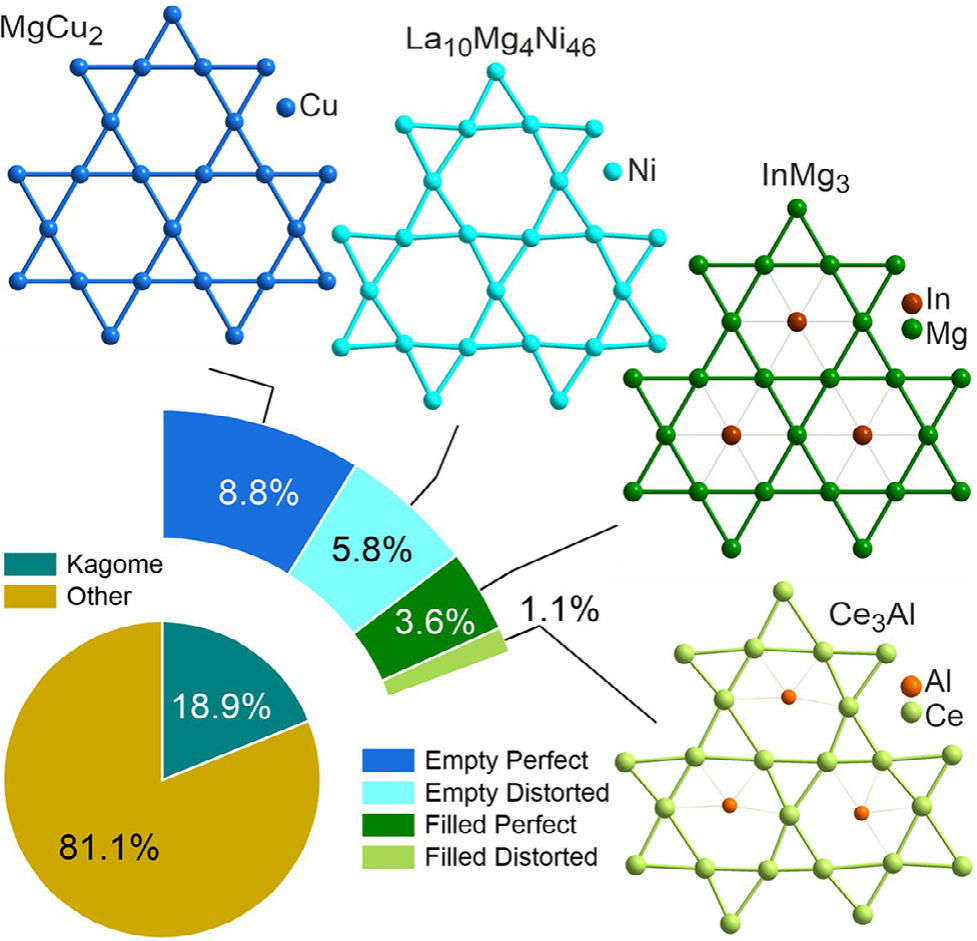
Percentage of intermetallic structures containing kagome layers (Table 1) and sub‐classification into the four classes. Example structures from each class are shown: MgCuZn_2_^[^ ^42^ ^]^ with perfect‐empty layers of zinc atoms; La_10_Mg_4_Ni_46_^[^ ^43^ ^]^ with distorted‐empty layers of nickel; InMg_3_^[^ ^44^ ^]^ with perfect kagome magnesium layers filled with indium; Ce_3_Al^[^ ^45^ ^]^ with distorted kagome layers of cerium filled with aluminum atoms.

The database is presented in the SI and contains unmerged entries so future users can determine criteria for merging according to their specific requirements. For the purposes of analysis and comparison, we have merged entries that had the same composition, space group and a difference in unit cell volume within 10%. The merged database contained 5749 entries with empty or filled kagome layers (see **Table** **1**). Analysis at the level of individual layers enabled the identification of structures that contained separate inequivalent layers; 189 entries contained more than one layer type that correspond to different kagome classes. Within all intermetallic compounds, 18.9% contain a single‐element kagome layer (**Figure** **3**). Of these entries, the majority (46.8%) correspond to perfect‐empty layers, followed by 31.0% of distorted‐empty ones. A further 19.4% of entries belong to perfect‐filled kagome and the remaining 6.0% correspond to distorted‐filled nets.

**Table 1:** Number of entries belonging to the four different classes in the database of kagome materials. Entries were merged as described in the text. 189 structures contain non‐equivalent layers corresponding to different classes of kagome; these entries were counted in each of the corresponding classes for the fractions listed.

	No. Entries	Fraction of intermetallics, %	Fraction of kagome, %
Intermetallics	30429		
Kagome layers	5749	18.89	
**Empty**	**4368**	**14.35**	**75.98**
Perfect	2693	8.85	46.84
Distorted	1783	5.86	31.01
**Filled**	**1388**	**4.56**	**24.14**
Perfect	1117	3.67	19.43
Distorted	346	1.14	6.02

**Figure 4:**
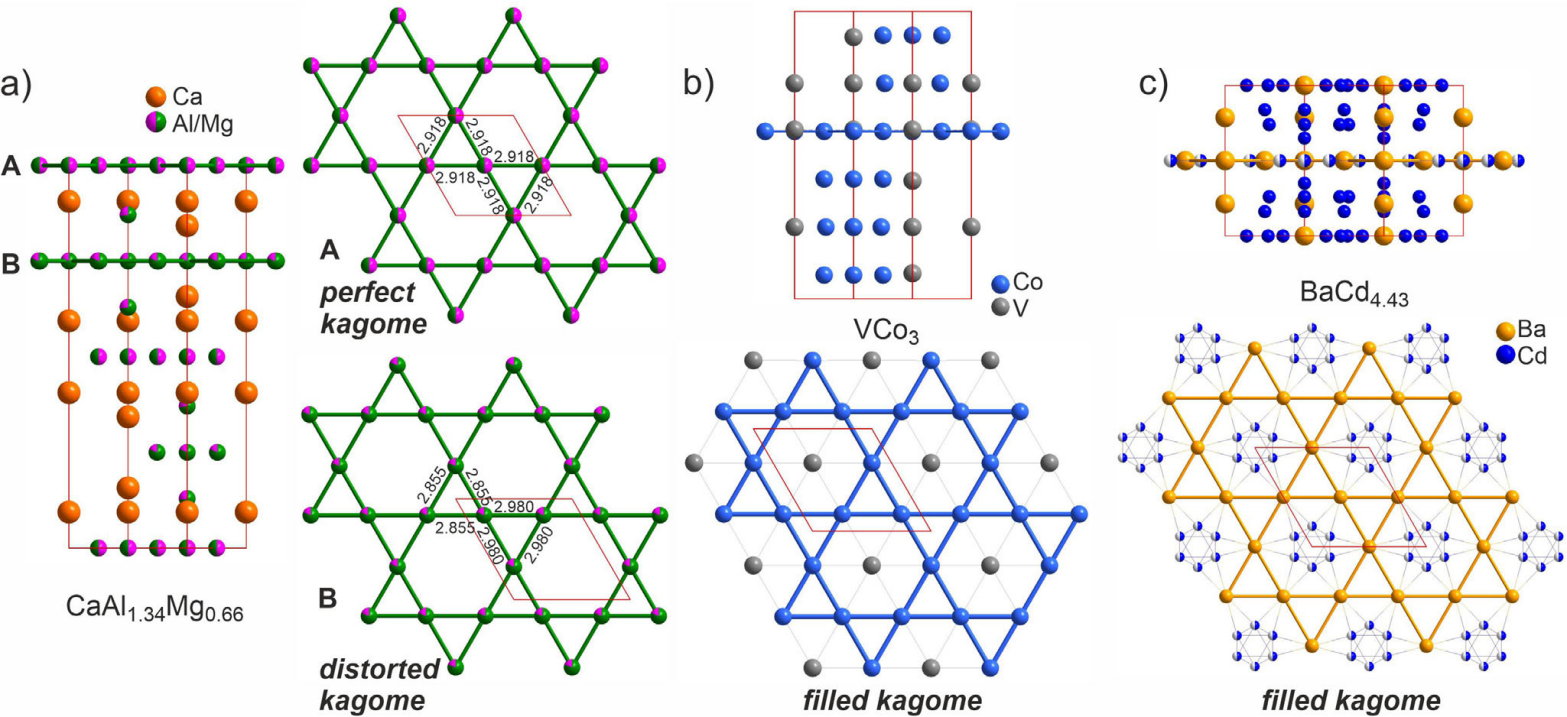
Examples of compounds that contain different classes of kagome layers. a) Structure of CaAl_1.34_Mg_0.66_ (MgNi_2_ structure type)^[^ ^48^ ^]^ with empty perfect (A) and slightly distorted (B) breathing kagome layers. b) Structure of VCo_3_^[^ ^52^ ^]^ with filled perfect kagome layers. c) Structure of BaCd_4.43_^[^ ^53^ ^]^ which contains kagome layers formed by barium atoms with hexagonal tiles filled with disordered cadmium sites.

The present database contains a comprehensive array of layers that are not limited by perfect geometry or their compliance with hexagonal symmetry. Such phases can still preserve topological flat bands despite having geometrically imperfect layers, as was demonstrated recently for YbTi_3_Bi_4_.^[^ ^46^ ^]^ Another benefit of combining topological and geometrical methods as implemented here is the ability to independently analyse individual layers within the structure. As a result, some entries in the database have multiple classifications because their structures feature multiple different types of kagome layers. For example, the MgNi_2_‐type Laves phases^[^ ^47^ ^]^ (illustrated by the example of CaAl_1.34_Mg_0.66_^[^ ^48^ ^]^ in **Figure** **4**a) support the coexistence of perfect (labelled A) and distorted (labelled B) kagome layers within a single structure. The choice of this entry from the CaAl_2‐x_Mg_x_ solid solution showcases significantly different bond lengths resulting in two types of triangles in the distorted layer (with bond length of 2.855 and 2.980 Å) distinct from the ones in the perfect layer (2.918 Å).^[^ ^49^ ^]^ This type of distortion is recognized as breathing kagome and is characterised with specific non‐trivial topological phenomena.^[^ ^50^ ^]^ Thus, the MgNi_2_‐type entries are labelled as both empty‐perfect and empty‐distorted simultaneously. Laves phases are considered to be classic examples of intermetallics with kagome layers;^[^ ^51^ ^]^ therefore, this detailed classification allows identification of distortions even within well‐known kagome structures.

The database also distinguishes between entries with filled and empty kagome layers. Filled kagome phases enable the discovery of flat bands and topological properties arising from appropriate orbital overlaps between the kagome layer and filled species.^[^ ^28^, ^34^ ^]^ One example of the structures that feature such layers is VCo_3_^[^ ^52^ ^]^ with a kagome arrangement of cobalt atoms filled by vanadium atoms (Figure 4b). At the same time, there are also some more unusual cases of filled kagomes such as the structure of BaCd_4.43_^[^ ^53^ ^]^ where half‐occupied cadmium hexagons fill the hexagonal tiles of kagome layers composed of barium atoms (Figure 4c). This demonstrates how the filtering algorithm identifies the topology of such a layer despite the complexity of bonding within it.

**Figure 5:**
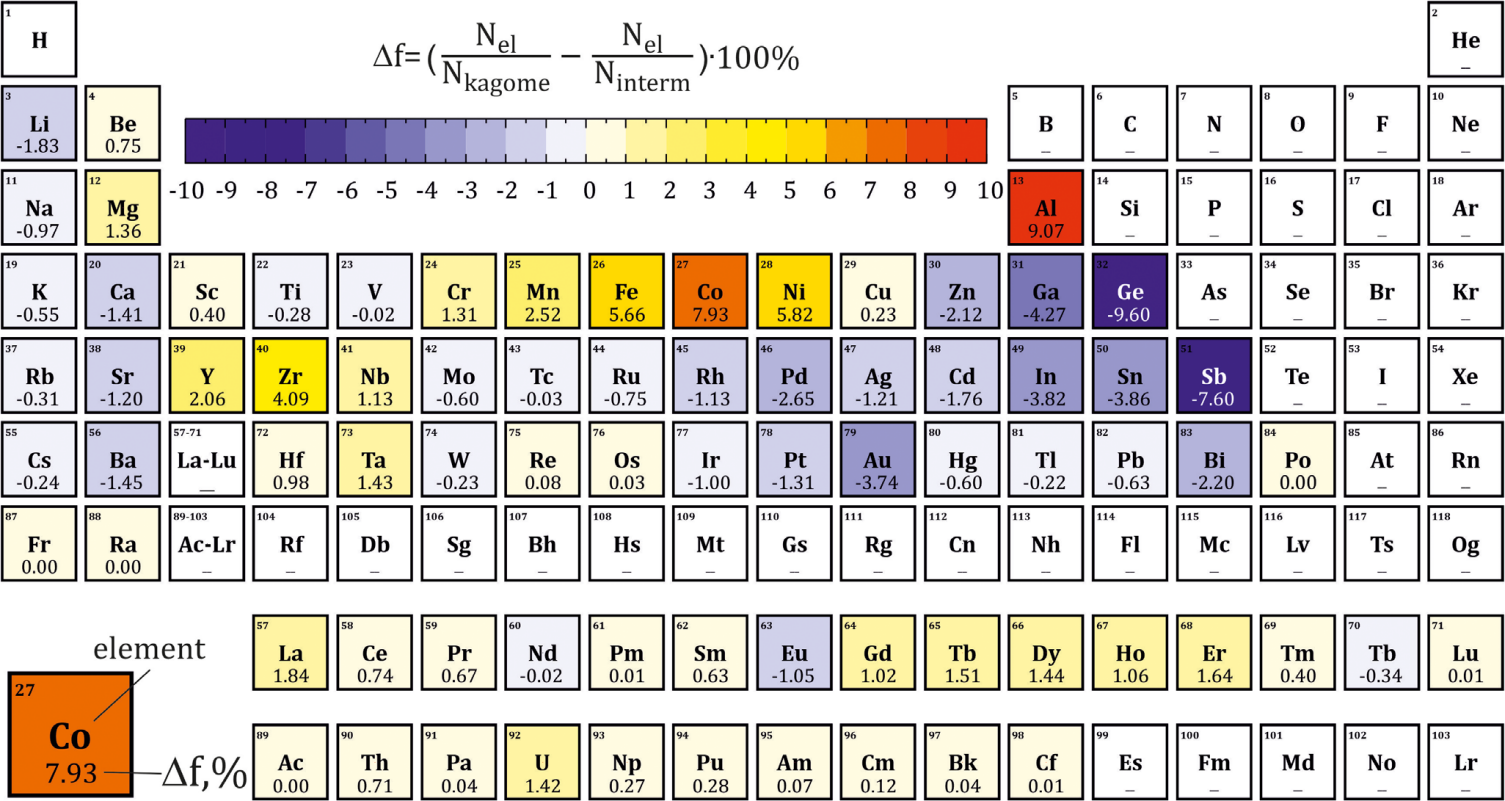
Difference (Δf) between occurrences of elements in the compounds within the kagome database compared to the entire database of intermetallics. Further insight is provided by analysis of the individual kagome classes (Figures S4–S7, Supporting Information).

Building the database with these classifiers enables us to identify some chemical trends. The analysis of the elements forming the compounds with kagome layers compared to the entire intermetallics database reveals a number of interesting tendencies (**Figure** **5**). While a prevailing number of Fe, Co, and Ni compounds were expected due to their well‐studied magnetic properties, the abundance of Al compounds in the database is less intuitive. Moreover, Ge and Sb compounds are noticeably less likely to form kagome layers than the other elements considered. This analysis was also made for the individual kagome classes (Figures S4–S7, Supporting Information) which revealed further insights into the chemistries that lead to these structures. The compounds with empty perfect layers mainly follow the trends found for all kagome compounds but show a preference for the rare‐earth elements, while phases with empty distorted layers are more likely to be found with Mg and Zr in their compositions. Filled layers have a slightly different distribution and show a higher frequency of entries with Pd (perfect layers) and Ti (both perfect and distorted).

**Figure 6:**
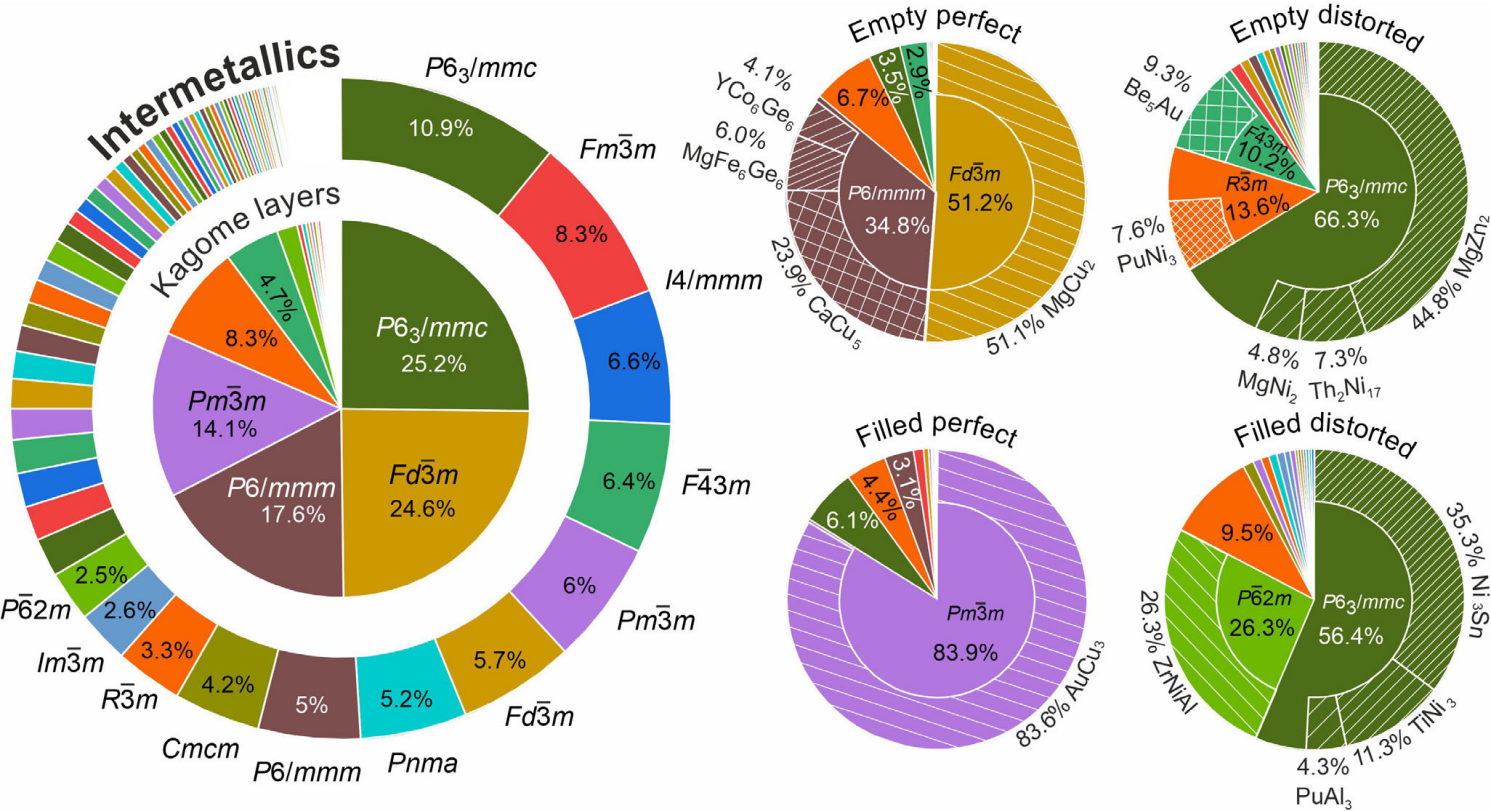
Space group distribution across entries with kagome layers compared with all intermetallics. The space groups corresponding to >2% and >10% of the entries are labeled for all intermetallics and kagomes, respectively. For the four types of kagome class, the most common (>4%) structure types corresponding to the dominant space groups are highlighted. The structure types are named as in the ICSD database. The lists of entries per space group and structure type are given in Tables S3–S8 (Supporting Information).

The perfect kagome layer should have the plane group *p*6*mm*. However, the individual layers are never isolated in a real 3D structure, and therefore the space group symmetry of the compound structure may be different from the plane symmetry of a specific layer. Furthermore, the symmetry of the layer can be lowered due to deviations from the geometrically ideal net. The distribution of space groups together with the corresponding plane group of the highest symmetry projection is given in Tables S3–S8 (Supporting Information). These data are aggregated and compared in **Figure** **6**, which shows that the majority of the kagome compounds belong to space groups consistent with the *p*6*mm* plane group: *P*6_3_/*mmc*, *Fd* $\bar{3}$ *m*, *P6/mmm*, and *Pm* $\bar{3}$ *m*.

Interestingly, the space group distribution is different within the four classes of kagome materials. The majority of empty perfect layers are represented by the cubic Laves phase structures (MgCu_2_,^[^ ^42^ ^]^ *Fd* $\bar{3}$ *m* space group). Further common structure types in this category are CaCu_5_^[^ ^54^ ^]^ (23.9%), MgFe_6_Ge_6_^[^ ^55^ ^]^ (6.0%), and YCo_6_Ge_6_^[^ ^56^ ^]^ (4.1%) which all adopt *P*6/*mmm* symmetry. A curious outlier here is the 3% of entries with the *F* $\bar{4}$3*m* space group, which corresponds to the *p*31*m* plane group, where the perfect geometry of the layers is preserved despite the loss of the sixfold axis. In contrast, filled perfect kagome are represented predominantly with the *Pm* $\bar{3}$ *m* space group (83.9% of these have the AuCu_3_^[^ ^57^ ^]^ structure type). Surprisingly, the majority of the distorted entries are still consistent with the *p*6*mm* plane group with *P*6_3_ */mmc* being the most common space group in both cases (66.3% for empty and 56.4% for filled kagomes). The hexagonal Laves phase MgZn_2_^[^ ^58^ ^]^ structure type is the most common for empty distorted layers (44.8%) while 35.3% of filled distorted kagome entries belong to the Ni_3_Sn^[^ ^59^ ^]^ structure family. At the same time, 26.3% of filled distorted entries belong to the ZrNiAl^[^ ^60^ ^]^ structure type with the *P* $\bar{6}$2*m* space group that is almost not represented among the other kagome classes. Interestingly, a recent study has identified a subset of entries with this structure type as potential candidates for unconventional superconductivity.^[^ ^32^ ^]^ In general, the distributions of space groups for the distorted classes is more diverse (see Tables S5 and S7, Supporting Information) with more rhombohedral, tetragonal and orthorhombic cases, consistent with symmetry breaking of parent cubic and hexagonal lattices. These results emphasise that the approach can reveal low‐symmetry kagome layers embedded in crystal structures with a diverse array of space groups, broadening the chemistries available to search for novel materials with these structural motifs.

### 2.3 Mapping Kagome Structural Classes onto Potential Physical Properties

**Figure 7:**
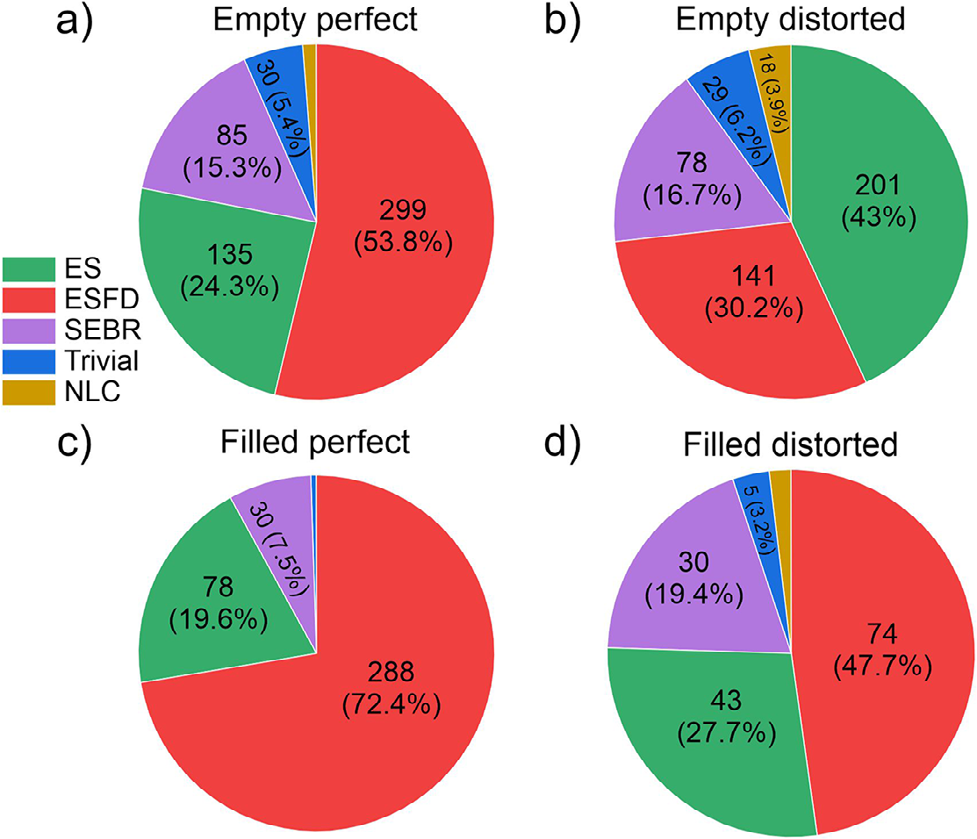
Distribution of topological materials properties across different classes of kagome: Enforced Semimetal (ES), Enforced Semimetal with Fermi Degeneracy (ESFD), Split Elementary Band Representation (SEBR), No Linear Combination of Elementary Band Representation (NLC), and Trivial topology in the band structures.^[^ ^61^ ^]^ The statistics shown are for the merged entries. The complete breakdown of the numbers of entries per kagome class is listed in Table S9 (Supporting Information).

To understand the relationship between the different structural classes of kagome materials and to illustrate how our method can be connected to other tools and databases to extract useful information about these materials and their potential physical properties, we have cross‐referenced the database produced here with entries in the Materials Flatband Database (MFD).^[^ ^30^ ^]^ From the full list of entries in the database, 51.4% (4921) are present in MFD, and only 2161 of these have been classified in MFD as containing a kagome net. We therefore ensure that every kagome‐containing entry in MFD can be appropriately labeled, connecting the four structural classes of kagome to the five topological materials properties of MFD. The arising distributions of topological material properties over the structural classes are shown in **Figure** **7**. An Enforced Semimetal (ES) represents a semimetal with gapless bands that do not satisfy the compatibility relations expected for a set of elementary band representations (EBRs) between high‐symmetry points, such as Na_3_Bi.^[^ ^61^ ^]^ Enforced Semimetals with Fermi Degeneracy (ESFD) are semimetals with gapless band crossings at the Fermi level, with HgTe being the prototypical example.^[^ ^62^ ^]^ Split Elementary Band Representation (SEBR) materials exhibit band topology similar to graphene, whose bands above and below the Fermi level can be expressed as linear combinations of EBRs (e.g., the bands arising from *p_z_* orbitals of graphene), whereas No Linear Combination of Elementary Band Representation (NLC) materials possess bands that cannot be linearly expressed in one or more EBRs and are physically similar to CaAs_3_.^[^ ^61^ ^]^ Materials with trivial topology are normal insulators whose bandgaps are not topological.^[^ ^61^ ^]^ More details are given in S3 (Supporting Information).

For those entries present in MFD, the distribution of ground‐state electronic structure topologies at the Fermi level differs for each structural class of kagome layer (see Figure 7), reflecting the connection between the structural layers in direct space and the band structure in reciprocal space. This shows how the chemistries that form the four different structural classes, reflecting the electron counts and bonding interactions that afford them, control the diversity of the different electronic ground states. For example, filled perfect nets are dominated by the metallic ESFD state, and have few trivial ground states, whereas empty perfect nets display the topological insulator NLC state that is missing only in their filled analogues among the four structural classes (see Table S9, Supporting Information). Chemistry affording the empty distorted net most frequently adopts the ES ground state, in contrast to filled distorted, where ESFD is most frequent, so filling the distorted kagome net changes the dominant electronic ground state. While the metallic ES and ESFD states dominate all structural classes, the fraction of topological insulators (NLC and SEBR) in both distorted classes is higher than in their perfect analogues, consistent with the associated distortions lifting some or all of the enforced degeneracies in those cases (see Figure 7; Table S9, Supporting Information).

By identifying the kagome net wherever it is found at scale, we are able to identify unexplored kagome‐containing topological materials and thus their associated structural families (details of the most common families are given in Table S10, Supporting Information) that are not labeled as kagome in MFD. Among the examples are two rhombohedral Ce_5_Co_19_‐type^[^ ^63^ ^]^ structures with empty perfect kagome layers, Pr_5_Ni_19_ and Nd_5_Ni_19_, that are labeled in MFD as ESFD and SEBR, respectively. There are 12 more structures in this structure type absent from MFD uncovered in our kagome database. Another example is Ti_3_Sn which exhibits a phase transition from *P*6_3_/*mmc* to *Cmcm* on cooling.^[^ ^62^ ^]^ Both structures contain filled distorted Ti‐kagome layers, with the symmetry lowering associated with a semimetal (ES) to TI (NLC) transition,^[^ ^61^ ^]^ demonstrating how engineering the symmetry within the net of a given compound dictates electronic structure properties. A further example is orthorhombic Ca_5_Bi_3_ (*Pnma*)^[^ ^64^ ^]^ with empty distorted layers which preserves the ES ground state despite having non‐hexagonal symmetry. Inspection of the band structures of Ca_5_Bi_3_ reveals degenerate flat bands and enforced nodal plane crossings at the Fermi level along the high‐symmetry paths.

The above examples, addressing all four of the non‐trivial topological electronic classes in MFD, emphasise the role of a general method in identifying the presence of the kagome net in any structure regardless of crystallographic symmetry, highlighting materials and associated structural families for more detailed investigation according to the research question of interest.

### 2.4 Machine Learning for Prediction of Kagome Intermetallics

We now apply the database in a machine learning (ML) setting to train two models to aid the experimental chemist in deciding which combinations of elements (phase field) to choose when targeting the discovery of new materials containing kagome layers, noting that the above analysis, connecting the chemistry affording the distinct net classes to the arising electronic properties, can be applied to novel kagome‐containing materials once identified. To assess the likely existence of new intermetallic materials within a particular phase field, we have used the established variational autoencoder (VAE) approach.^[^ ^65^, ^66^, ^67^ ^]^ A VAE was trained using all 1039 phase fields containing an entry in the intermetallics dataset extracted from the ICSD (Section S4, Supporting Information) as the ground truth dataset and removing all elements with an atomic number above 87. A query dataset was then generated containing the 454087 quaternary phase fields which contain the combinations of four individual elements within the ground truth dataset and removing combinations present in the ground truth dataset. These represent potentially unexplored quaternary intermetallic phase fields. The maximum fraction difference (MFD)^[^ ^68^ ^]^ metric was used to identify an optimal threshold in the reconstruction error of the VAE and to assess the performance of this model in identifying phase fields more likely to contain undiscovered quaternary intermetallic compounds. For the best performing VAE (SI4) that uses magpie features to represent elements compressed to a 6‐D latent space, the MFD was 0.47, in line with well performing VAE models for the task of proposing novel metal‐organic framework chemistries.^[^ ^68^ ^]^ A total of 94, 039 phase fields from the query dataset had reconstruction errors below the optimal threshold of 3.6 and are taken forward as potential phase fields containing novel quaternary intermetallics.

To identify which of these phase fields are likely to contain compounds with kagome layers in their crystal structures, a binary classifier was trained using the 150 quaternary phase fields containing an entry in the database labelled as containing a kagome layer as positive training data, and the remaining 889 quaternary phase fields present in the intermetallics database as negative training data. The best performing model (SI4) was built using magpie feature vectors to represent each element and a boosted decision tree architecture, having a Matthew's correlation coefficient of 0.74 based on a 10% hold‐out set. The probability of classification arises naturally from the boosted decision tree architecture, and is hereafter referred to as the kagome probability (technical details in S4, Supporting Information).

Of the 94, 039 quaternary intermetallic phase fields with VAE reconstruction errors below the optimal threshold, 4462 were classified as positive (likely to contain at least one compound with a kagome layer in its crystal structure) by the binary classifier. To further guide the experimental chemist, a Pareto front was built from the VAE reconstruction error and the probability of a positive label taken from the binary classifier, where low VAE reconstruction errors and high probabilities represent unexplored quaternary intermetallic phase fields that are likely to contain a compound that is both novel and contains a kagome layer in its crystal structure. The first Pareto front contained 17 phase fields (see **Table** **2**). Further investigation revealed that three of these (Ni‐La‐Ce‐Mg, Mn‐Ni‐La‐Mg, Mn‐Ni‐Zr‐Ti) contained compounds^[^ ^70^, ^71^, ^72^, ^73^, ^74^, ^75^ ^]^ displaying kagome layers in their crystal structures that are present in the Pearson's Crystal Dataset^[^ ^69^ ^]^ but not in the ICSD^[^ ^39^ ^]^ – these compounds are therefore not present in the training set. These can be considered as successful “hits” for the VAE and binary classifier models.

**Table 2:** Phase fields that were found to lie on the first Pareto front based on the probability that they contain at least one phase with kagome layers and their reconstruction errors achieved by the autoencoder (details in SI4). Quaternary phases reported to contain kagome layers in the Pearson's Crystal Dataset^[^ ^69^ ^]^ that are absent from ICSD^[^ ^39^ ^]^ which was used in training the models.

Phase Field	Kagome Probability	Reconstruction Error	Hits in Pearson's Crystal Dataset
Ni Mg Pr Y	1.00	3.36	0
Co Ni La Gd	1.00	3.29	0
Ni La Ce Mg	1.00	3.27	MgLa_0.5_Ce_0.5_Ni_4_^[^ ^70^ ^]^
Co Ni La Y	1.00	3.22	0
Ni Al Pr Mo	1.00	3.19	0
Ni Ca Sc Y	1.00	3.18	0
Ni La Li Cr	1.00	3.06	0
Co Ni Mg Ho	1.00	3.02	0
Ni Li Mg Gd	1.00	2.97	0
Ni Sc Mg Gd	1.00	2.92	0
Ni Ti Nb Hf	1.00	2.82	0
Mn Ni La Mg	1.00	2.76	MgLaMn_0.3_Ni_3.7_,^[^ ^71^ ^]^ MgLaMn_0.5_Ni_3.5_, MgLaMnNi_3_^[^ ^72^ ^]^
Mn Sc Sn Ga	1.00	2.61	0
Ni Ti Sc Nb	1.00	2.42	0
Mn Ni Zr Ti	1.00	2.31	Zr_0.05_Ti_0.95_Mn_1.5_Ni_0.5_,^[^ ^73^ ^]^ Zr_0.05_Ti_0.95_Mn_1.45_Ni_0.5_,^[^ ^74^ ^]^ Zr_0.8_Ti_0.2_Mn_0.7_Ni_1.3_^[^ ^75^ ^]^
Ni Ti Nb V	0.989	2.27	0
Ni Ti Cr V	0.531	2.22	0

The machine learning workflow can highlight chemistry that leads to materials with interesting physical properties based on the kagome layer. This can be exemplified with compounds that are not present in the training data and are identified within such top‐ranked phase fields. In addition to the phase fields highlighted above, the first Pareto front also features the Mn‐Sc‐Sn‐Ga phase field, which has a high kagome probability of 1 (Table 2) and does not correspond to any entry in the ICSD and Pearson databases. This ML workflow guided the identification of the compound ScMn_6_Sn_5.8_Ga_0.2_^[^ ^76^ ^]^ that belongs to the MgFe_6_Ge_6_ structure type (*P*6/*mmm* space group) and contains empty perfect kagome layers of Mn atoms together with a disordered Sn/Ga net. This material has been experimentally characterized in 2004 with a temperature‐dependent ferro‐to‐helimagnetic transition, suggesting a competition between ferro and antiferromagnetic interactions that can be attributed to the frustration inherent to the kagome net.^[^ ^76^ ^]^

Another quaternary phase field presenting a high kagome probability in the fifth Pareto front (Table S12, Supporting Information) that does not correspond to any entry in the ICSD database is Er‐Mn‐Sn‐Ge. In this case, the model guided the identification of a compound present in the Pearson database with the composition ErMn_6_Sn_4_Ge_2_.^[^ ^77^ ^]^ It is related to the well‐studied ternary kagome ErMn_6_Sn_6_^[^ ^78^ ^]^ with a Mn empty perfect kagome net. Unlike ScMn_6_Sn_5.8_Ga_0.2_, here the Sn and Ge are ordered over the original Sn positions. Despite not affecting the structure of the magnetic net, this ordering directly influences the magnetic properties compared to the parent compound, with a higher ferri‐to‐antiferromagnetic ordering temperatures and higher magnetic susceptibilities.^[^ ^77^, ^78^ ^]^

In addition, more subtle changes in the magnetic anisotropy of ErMn_6_Sn_4_Ge_2_ are expected compared to that of ErMn_6_Sn_6_^[^ ^76^ ^]^ where the alignment of the magnetic moments can be tuned along the *c*‐axis and *ab*‐plane for a wide range of temperatures.^[^ ^79^ ^]^ Controlling the magnetic anisotropy in kagome magnets and achieving perpendicular magnetic anisotropy is important for integration in spintronic devices,^[^ ^80^ ^]^ while the influence of the magnetic states on the electronic properties is uncharted.^[^ ^79^ ^]^ This is exemplified in the calculated electronic structures of the two magnetic states in ErMn_6_Sn_4_Ge_2_, which show that the *c*‐axis ferrimagnetic state exhibits linear Dirac dispersions around the high‐symmetry point near the Fermi level, while the *ab*‐plane ferrimagnetism shows more parabolic dispersions around the same point (see S5, Supporting Information), which originate from the *d* orbitals of the kagome Mn atoms, suggesting higher mobilities for uniaxial anisotropy.

These two separate examples show that the exploration of the phase fields from the workflow will enable us to identify new phases containing perfect kagome nets located within distinct ordered or disordered environments that give access to a range of electronic and magnetic structures that can be tuned for specific applications. This ML workflow is applicable to nets beyond kagome given the construction of databases for such nets.

## 3 Conclusion

We present a comprehensive database of kagome‐containing intermetallics by evaluating all candidates according to topology then geometry, separating the symmetry of the net itself from that of the solid it forms part of and retaining disordered examples, including those where the disorder is in another component of the structure. This maximises information about the connection between chemistry and kagome net formation, demonstrated by the construction of an ML model that identifies known kagome‐forming chemistries that were not present in the training data used. Capturing all recognisable kagome nets enables classification into perfect and distorted examples. This allows identification both of the physics associated with the distortions and of those examples that retain important symmetries for transport and magnetic properties despite their imperfection, and highlights candidates for chemical or external field transformation from one geometry to another. The database can be tailored to afford more focussed lists that address specific research questions. For example, correctly labelling the kagome net wherever it is found independent of parent structure crystallographic symmetry identifies unexplored families of kagome‐based solids as candidates for topologically protected electronic properties. The sorting workflow can be applied to other classes of materials and structural fragments of interest such as regular (honeycomb, square planar, triangular) and semiregular plane nets,^[^ ^13^ ^]^ and 3D networks.

## Conflict of Interest

The authors declare no conflict of interest.

## Supporting information



Supporting Information

Supporting Information

## Data Availability

The tools for generating the database and the machine learning models are available on Github repository: https://github.com/lrcfmd/kagome_database.
