# Supplementary material for: Topology Augmented with Geometry in the Assembly of Structural Databases: Kagome Intermetallics
Source: Adv Sci (Weinh). 2025 Jul 4;12(35):e17041. doi: 10.1002/advs.202417041 (PMC12463092; doi:10.1002/advs.202417041)
Supplement: Supplementary file 1 — Supporting Information [file ADVS-12-e17041-s001.pdf]

ADVANCED SCIENCE
Open Access

Supporting Information

for *Adv. Sci.*, DOI 10.1002/advs.202417041

Topology Augmented with Geometry in the Assembly of Structural Databases: Kagome Intermetallics

*Nataliya L. Gulay, Dongsheng Wen, Joshua E. Griffiths, Judith Clymo, Luke M. Daniels, Jonathan Alaria, Matthew S. Dyer, John B. Claridge and Matthew J. Rosseinsky**

Topology augmented with geometry in the assembly of structural databases: kagome intermetallics

Authors:

Nataliya L. Gulay[1+], Dongsheng Wen[1+], Joshua E. Griffiths [1], Judith Clymo[2], Luke M. Daniels[1], Jonathan Alaria[3], Matthew S. Dyer[1], John B. Claridge[1], Matthew J. Rosseinsky[1,4]*

1. Department of Chemistry, University of Liverpool, Materials Innovation Factory, 51 Oxford Street, Liverpool, L7 3NY, UK

2. Department of Computer Science, University of Liverpool, Ashton Street, Liverpool, L69 3BX, UK

3. Department of Physics, University of Liverpool, The Oliver Lodge Laboratory, Liverpool, L69 7ZE, UK

4. Leverhulme Research Centre for Functional Materials Design, Materials Innovation Factory, 51 Oxford Street, University of Liverpool, Liverpool L7 3NY, UK.

[+] *These authors declare equal co-first authorship based on equal contribution to the paper*

Supplementary information

Table of contents:

SI1	Methods for Building the Database	2
SI2	Additional Data	5
SI3	Details on topological characterisation	14
SI4	Detailed Methods of ML Classification and Chemical Similarity Ranking	17
SI5	Band structure calculations for $ErMn_6Sn_4Ge_2$	27
	References	30

Si1. Methods for Building the Database

1.1. Starting database

For a starting database of intermetallic compounds, the entries from the International Crystal Structure Database (ICSD)[1] were selected which were composed of at least two metallic elements (categorized according to *Steurer and Dshemuchadse* in the *Intermetallics: Structures, Properties and Statistics,*[2] see Figure SI1 for a full compositional range). No further restrictions were applied at this stage. The initial database consisted of >40,000 entries.

Obtained entries were processed using the custom code SplitCif.py which produces the crystallographic files for each of consisting chemical species enabling search of single-element kagome layers. Analysis of such individual sublattices allows to search for the kagome layers regardless of the further connectivity which simplifies interpretation of the adjacency matrices (discussed below). This step is also consistent with previous works.[3] Resulting list of single-element substructures contained >110,000 entries. This database was used for further topological and geometrical filtering.

1.2. Topological screening

The first stage of analysis was performed using the software ToposPro (v. 5.5.1.0)[4,5], which enabled screening of a database for the structural element of interest. At the start, the adjacency matrices, which define the connectivity of an atom with adjacent ones within the coordination shells, were calculated for all single-element entries using the ADS tool of the software suit. For this, the Voronoi-Dirichlet partitioning[6] was performed for each independent atom in the structure and the information on the connectivity was recorded numerically in a form of graph.[7] The suitable planar fragment (root-mean-square deviation from the RMS plane within 0.1 Å) of a kagome topology was saved and used to identify the entries which contain it. Higher plane deviation threshold yields more matches (e.g. ~2,000 entries if set up to 0.5 Å, see Table SI1) but also lists entries that have strong deviations from a plane shown in Figure SI2. Therefore, the smaller threshold of 0.1 Å was chosen for creation of the database. The search of the kagome layer can be realized through searching the subnets within the supernets in the periodic crystal structures.[8] The supernet can be consider as the graph formed by all the nodes (atoms) and edges (bonds) in the structure. Similar to graph-subgraph relation, a subnet of the supernet is another net formed from a subset of the nodes and edges of the supernet.[8] As illustrated in Figure SI3, the software will unavoidably find both the lattice of interest (kagome in our case) and its higher symmetry supernets (hexagonal in our case), as well as various distorted layers. Therefore, we have decided to perform additional search for hexagonal layers and subtract entries containing them. Suitable reference fragments for kagome and hexagonal layers of various size were saved as graph files and used for further filtering. Using these fragments and applying the planar restriction, we have performed a search for compounds containing kagome and hexagonal layers and saved them in separate lists. Subtracting the latter from the former, the database containing only topological kagome layers was created.

After the initial topological analysis in ToposPro, the layers of interest were extracted as *.gph* files (containing the atomic positions of the identified fragment) for further geometrical evaluation.

1.3. Geometrical filters

Geometrical filters can access the chemical and bonding environment necessary for the further analysis, enabling the classification of supernets and distorted structures. Using the *Pymatgen* package[9] we have developed an algorithm to process ToposPro's output files and quantify the distortion degree based on the variations of the first-nearest neighbor (1NN) bond lengths and angles,

see Figure 2, b. This process provides (1) an accurate filter for the kagome layer identified through the topological method, and (2) quantifications of distortions of the kagome layers. It was noticed that the graph files (files with a suffix of *.gph*) (with only atomic site coordinates) from ToposPro may exclude atoms during subgraph construction and do not maintain periodic boundary conditions. Therefore, the potential kagome plane of a structure was identified by mapping the atomic sites of the original *.cif* file onto the plane defined by the *.gph* files from ToposPro using the algorithms built upon functionalities offered in the *Pymatgen* package.[10] The plane structure contains the atoms in the original crystal structure, and was saved as a new *.cif* file for structural analysis.

The 1NN environments of an atomic site were identified using the CrystalNN module implemented in *Pymatgen*.[10] Considering the compositional disorder of a site, the 1NN search starts with $r_i = \sum_s c_s r_s$, where r_i represent the atomic radius of the species-*s* on the site *i* and c_s represent the concentration of the species *s* on site *i*. We noticed that the CrystalNN module could potentially detect false 1NN information[10] based of the input structures and consequently lead to false calculations of the bond lengths and angles; therefore, the 1NN search cutoff radius was set for 7 Å to include more atomic bonds for CrystalNN to determine the 1NN of the atoms that form the layer based on the normalized Voronoi weights [10]. We found that this setting can minimize the false calculations of bond lengths and angles for the distortion analysis below. Since only one layer is selected for 1NN analysis, out-of-plane coordination was excluded. A geometrical filter for empty kagome is imposed by the condition that all atoms within the plane have a coordination number of four (Figure 2, *b*). For filled kagome layers, the sites within the hexagons are occupied by different species, different from the hexagonal layers with the same species occupying the hexagon centres. If the distances from the atoms to the identified kagome plane are within 0.15 Å, the atoms are considered as filled atoms of the hexagon. This allows exclusions of severely distorted nets where CrystalNN detected unreasonable in-plane coordination.

A python program was developed to parse all the atomic sites and measure the bond lengths and angles around 60˚ or 120˚ (as schematically shown in Figure 2,b), which will be used to quantify the degree of distortions of the atomic plane. Here we define three metrics of the lattice plane: $\delta(\{\theta_i\})$, $\delta(\{\varphi_i\})$, and $\delta(\{d_i\})$. $\delta(\{\theta_i\})$ and $\delta(\{\varphi_i\})$ are the normalized standard deviation of the triangle angles around 60˚ and hexagonal angles around 120˚, respectively, for each atoms on the plane:

$$\delta(\{\theta_i\}) = \frac{\sigma(\{\theta_i\})}{\mu(\{\theta_i\})}, \theta_i \in (40^\circ, 80^\circ)$$

$$\delta(\{\varphi_i\}) = \frac{\sigma(\{\varphi_i\})}{\mu(\{\varphi_i\})}, \varphi_i \in (80^\circ, 160^\circ)$$

where $\{\theta_i\}$ is an array of angles of the triangles within $\pm 20^\circ$ range of 60˚ (or $\{\varphi_i\}$ for angles of the hexagons within $\pm 40^\circ$ range around 120˚) for every atomic site-*i* on the kagome layer. σ() and μ() are the standard deviation and mean values of the angles, respectively. Similarly, for each angle θ_i, the first nearest bond length opposite to the angle is measured as d_i, which will be used to compute the normalized standard deviation of the bonds $\delta(\{d_i\})$:

$$\delta(\{d_i\}) = \frac{\sigma(\{d_i\})}{\mu(\{d_i\})}$$

where $\sigma(\{d_i\})$ and $\mu(\{d_i\})$ are the standard deviation and mean of the bond lengths. The normalization results in unitless quantities, enabling the comparison between different cell structures and atomic species. The above metrics were used to distinguish between perfect and distorted layers. For perfect layers, all 1NN bonds are equivalent, and all φ_i and θ_i are exactly 60˚ and 120˚; therefore,

$\delta(\{d_i\})$. $\delta(\{\theta_i\})$ and $\delta(\{\varphi_i\})$ are zero. These metrics are non-zero for slight distortions on the layer, enabling the separation of perfect and distorted layers. For the filled kagome layers, the atoms at the centres of the hexagons are removed before measuring the bond lengths and angles such that distortions are quantified for atoms that form the kagome layers. If the averaged angles ($\mu(\{\theta_i\})$) deviate from 60˚ by 10˚ or more, the layer is likely severely distorted and classified as non-kagome;[3] therefore, layers above the threshold are identified as non-kagome in this study. Distortion thresholds allow users to filter specific distortions and yield various dataset for various needs and interests. As a results, the structures are labelled based on the class of kagome layers (empty/filled) and the distortions of each class (perfect/distorted), see Figure 2(a).

1.4. Data Analysis

Difference Δf between occurrences of elements in the compounds within the kagome database compared to entire database of intermetallics was calculated using the following formula:

$$\Delta f = \left(\frac{N_{el}}{N_{kagome}} - \frac{N_{el}}{N_{interm}}\right) \cdot 100\%$$

Where N_{el}, N_{kagome}, and N_{interm} are the number of entries with an element, the number of entries in the kagome database, and the number of entries in the intermetallics database, respectively.

SI2. Additional Data

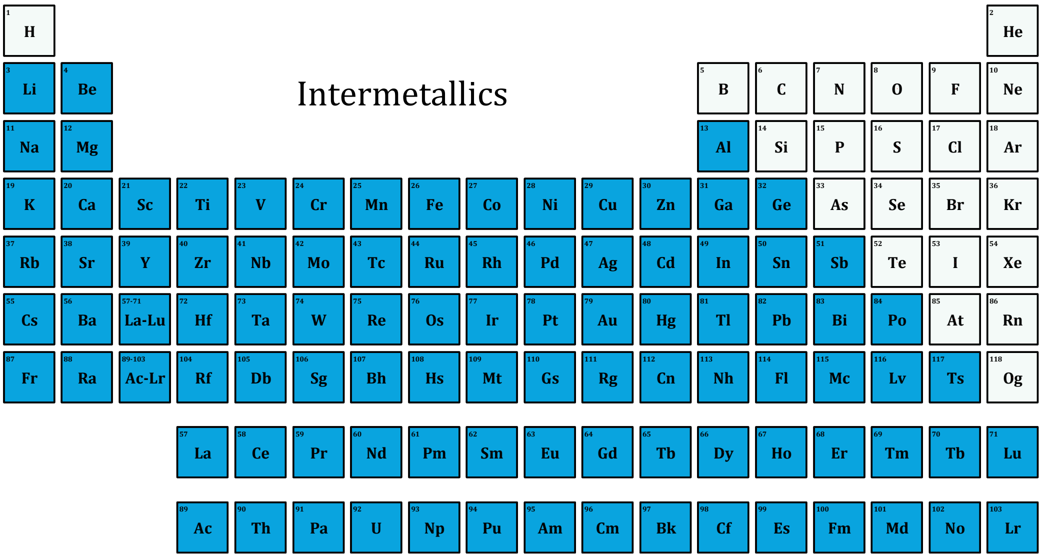

Figure SI1. Elements selected in the database. The classification of intermetallic compounds is made according to *Steurer and Dshemuchadse* [2].

Table SI1. Influence of planar threshold on the number of entries produced by ToposPro. using the single-element files created by SplitCif. Example structures with σ > 0.1 are shown in Figure SI2.

Deviation from the RMS plane (σ), Å	Number of entries found in single-element intermetallics database	Difference in entries found
0.01	20654	-178
**0.1**	**20832**	**0**
0.2	21440	+608
0.5	22821	+1989

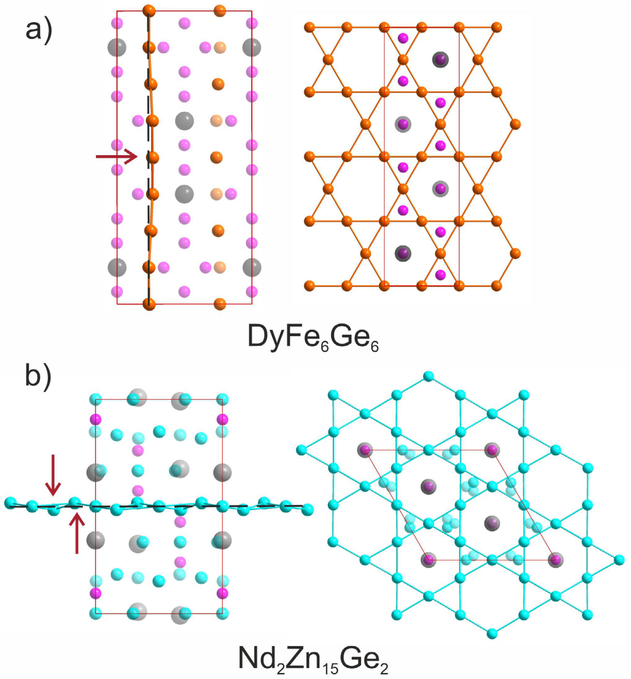

Figure SI2. Example structures that have kagome topology but are excluded from the database because of the plane deviation threshold σ > 0.1 Å. a) The structure of $DyFe_6Ge_6$[11] is included if threshold is set to σ = 0.2 Å. A number of entries based on this structure type ($TbFe_6Sn_6$[12]) meet the plane deviation threshold of σ < 0.1 Å so are included in the database. b) $Nd_2Zn_{15}Ge_2$[13] entry found if threshold is σ = 0.5 Å which has a distorted kagome layer with strong deviations from the plane.

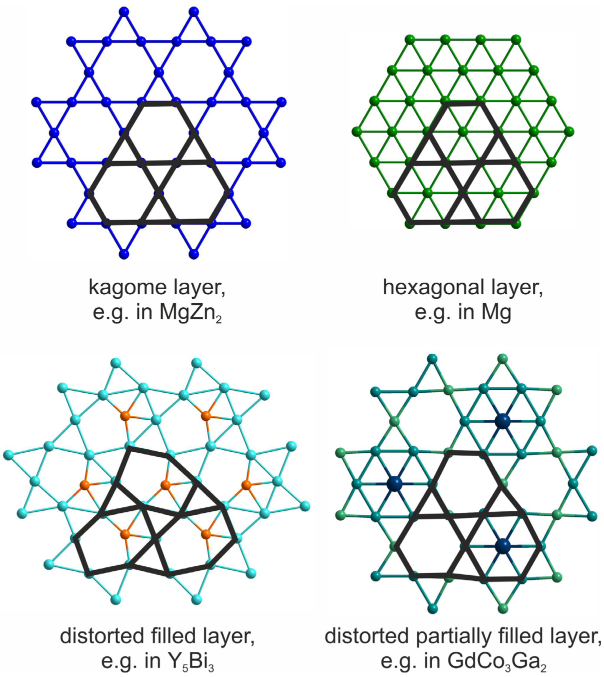

Figure SI3. Examples of structures containing layers which can be topologically recognized as kagome by ToposPro. $MgZn_2$[14] ($P6_3/mmc$) with kagome layer, Mg[15] ($P6_3/mmc$) with close-packed net, and Y_5Bi_3[16] (*Pnma*) and $GdCo_3Ga_2$[17] (*P*6/*mmm*) with different distorted topologies.

Table SI2. Comparison of the outcomes of recent publications that identify compounds with kagome layers. Starting database, summary of methodology as stated in the source publication, and final number of kagome entries are listed. The number of intermetallic entries that contain kagome layers in each study is highlighted in bold in the bottom row. The comparison was made for unique entries (i.e., entries are merged by composition and space group, see SI1 for this study and the individual methodologies for the previous works).

Study	**Meschke et al.** [18] **(2021)**	**Regnault et.al.** [19] **(2021)**	**Chiu et al.** [20] **(2022)**	**Jovanovic et. al** [21] **(2022)**	**Neves et al.**[3] **(2024)**	**This work**
Source databases	ICSD + AMCSD	ICSD, TQCDB [22]	Materials Flatband Database	ISCD	Materials Project	ICSD
Number of initial entries	~40,000 + ~13,000	55,206	55,206	96,233	139,367	43,164
Compositional restrictions	Main-group containing	No lanthanides (except for La) or actinides	No restrictions	No restrictions	No restrictions	Intermetallics
Methods	Geometric search on Wyckoff positions + further DFT of selected subset	Space group and geometrical methods search for sublattices	Geometric method based on the connectivity of sites	Geometric check for distances and angles	Geometric check for nearest neighbour distances	Topological screening (ToposPro) followed by geometrical filters
Kagome entries found (**intermetallic**)	498 (**102**)	6,120 (**443**)	1,331 (**337**)	3,484 (**1,771**)	5,752 (**451**)	5,564 **(5,564)**

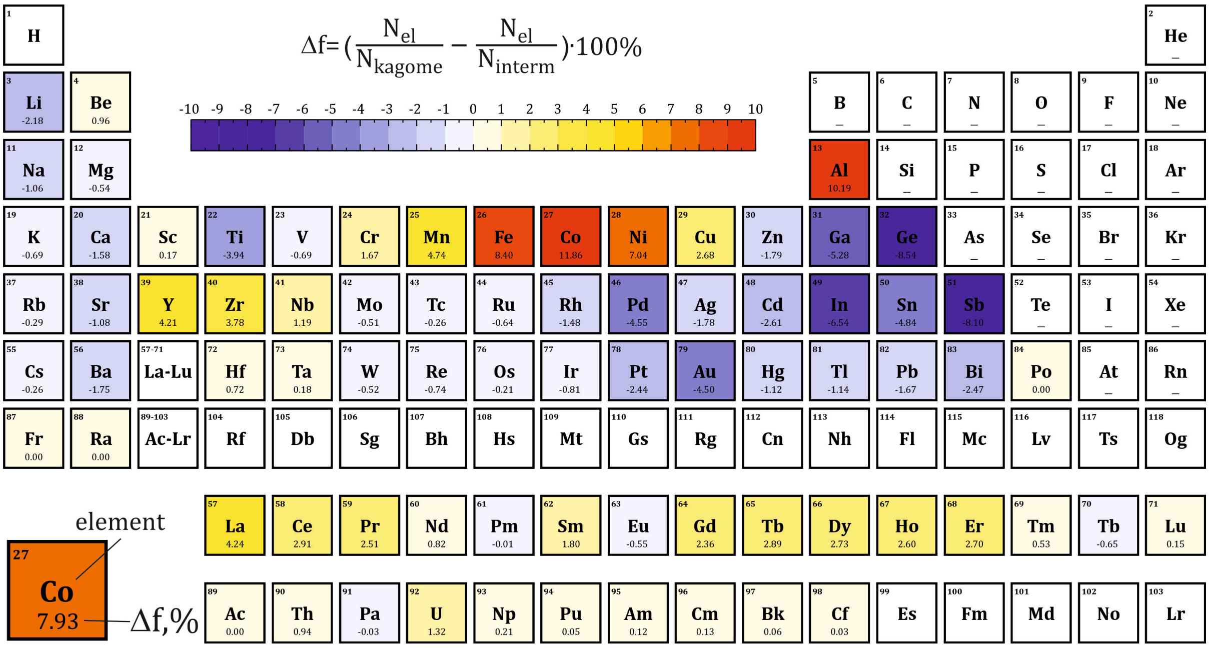

Figure SI4. Difference Δf between occurrences of elements within the empty perfect kagome class compared to entire database of intermetallics. The values are calculated as described in SI1.4.

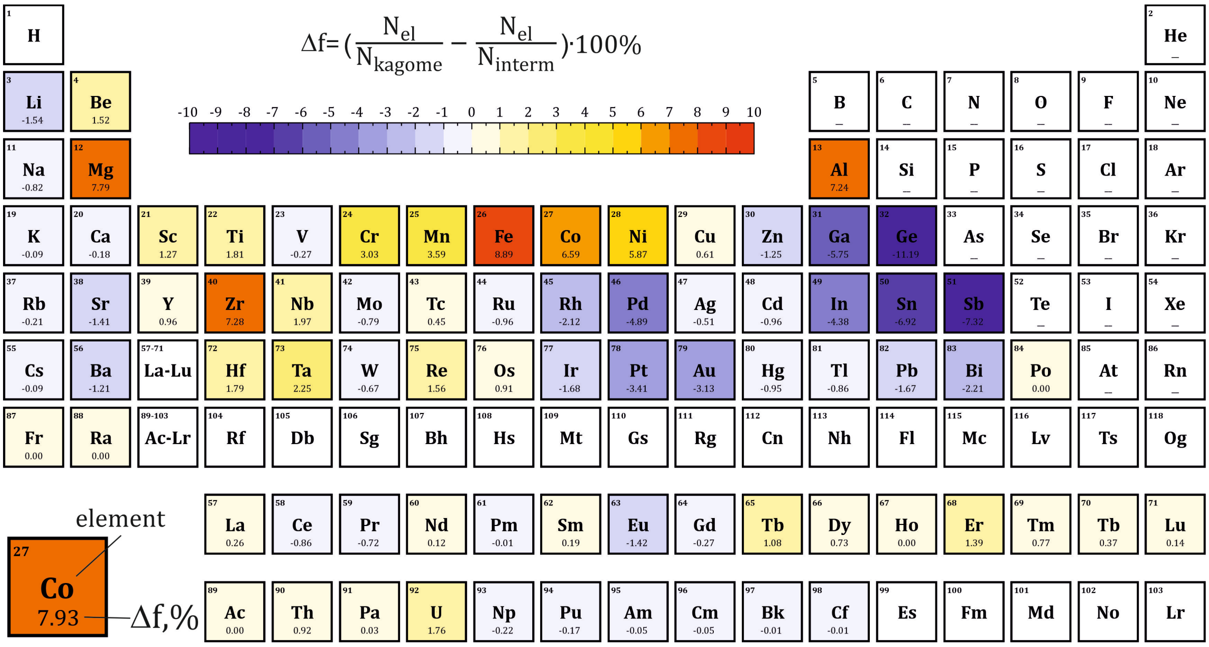

Figure SI5. Difference Δf between occurrences of elements within the empty distorted kagome class compared to the entire database of intermetallics. The values are calculated as described in SI1.4.

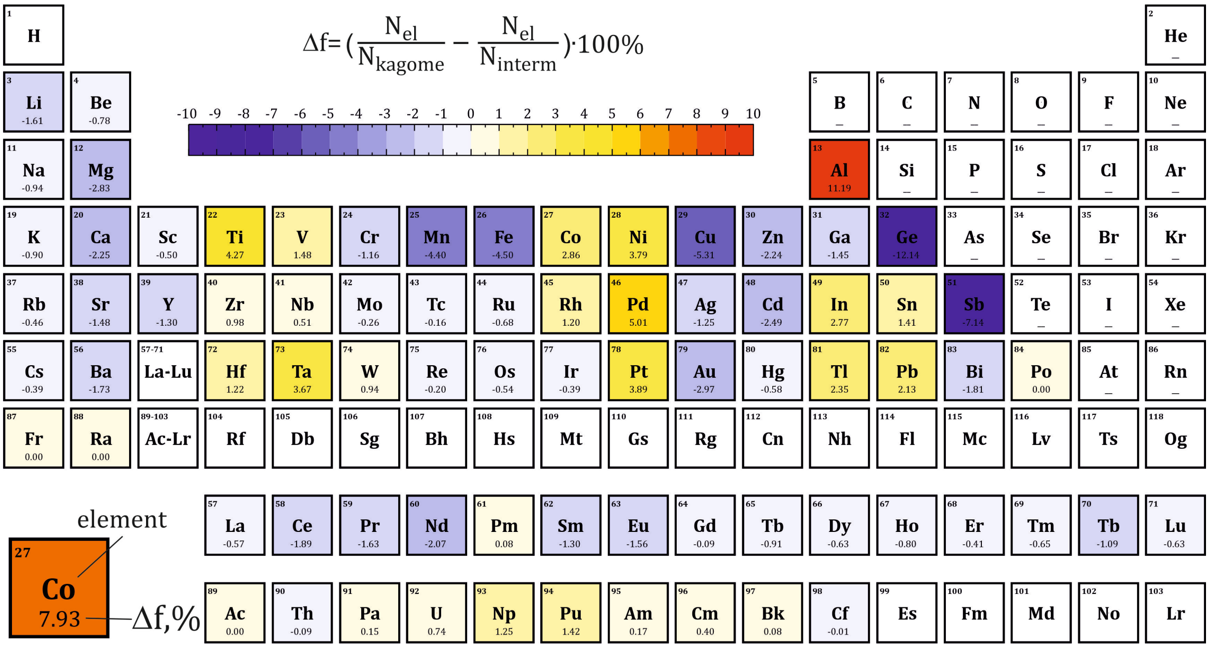

Figure SI6. Difference Δf between occurrences of elements within the filled perfect kagome class compared to the entire database of intermetallics. The values are calculated as described in SI1.4.

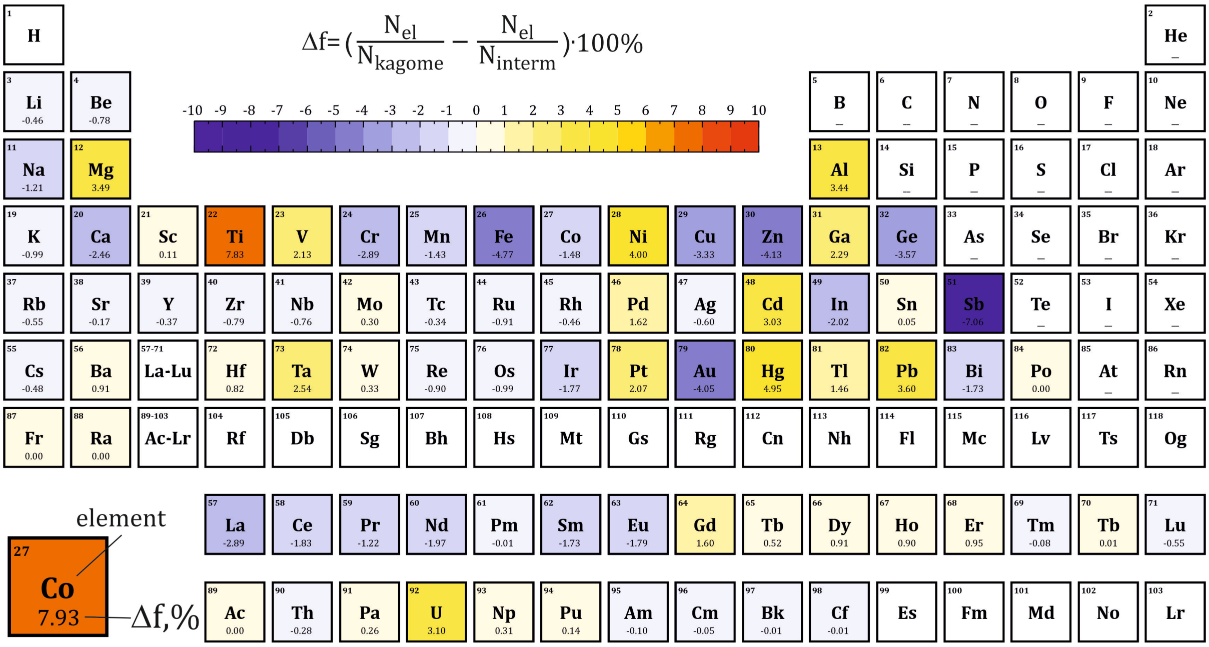

Figure SI7. Difference Δf between occurrences of elements within the filled distorted kagome class compared to entire database of intermetallics. The values are calculated as described in SI1.4.

Table SI3. Space group distribution across the total kagome database and the different kagome classes. The space group number, space group symbol, number of entries and the fraction corresponding to the number of those entries in each specific class are listed. The space groups are sorted in descending order of their fraction of the database of intermetallic materials extracted from ICSD. Space groups with fraction >1.0% are listed.

Space group		Intermetallics		Kagome layers		Empty perfect		Empty distorted		Filled perfect		Filled distorted	
		Entries	Fraction, %	Entries	Fraction, %	Entries	Fraction, %	Entries	Fraction, %	Entries	Fraction, %	Entries	Fraction, %
194	$P6_3/mmc$	3320	10.91	1402	24.39	93	3.45	1182	66.29	68	6.09	195	56.36
225	$Fm\bar{3}m$	2515	8.27	12	0.21	5	0.19	0	0.00	12	1.07	0	0.00
139	$I4/mmm$	1999	6.57	5	0.09	0	0.00	5	0.28	0	0.00	1	0.29
216	$F\bar{4}3m$	1935	6.36	261	4.54	79	2.93	182	10.21	0	0.00	0	0.00
221	$Pm\bar{3}m$	1840	6.05	786	13.67	0	0.00	0	0.00	937	83.89	0	0.00
227	$Fd\bar{3}m$	1731	5.69	1367	23.78	1379	51.21	2	0.11	6	0.54	0	0.00
62	$Pnma$	1587	5.22	2	0.03	0	0.00	1	0.06	0	0.00	1	0.29
191	$P6/mmm$	1516	4.98	981	17.06	938	34.83	17	0.95	35	3.13	0	0.00
63	$Cmcm$	1284	4.22	15	0.26	0	0.00	11	0.62	0	0.00	4	1.16
166	$R\bar{3}m$	1007	3.31	460	8.00	181	6.72	242	13.57	49	4.39	33	9.54
229	$Im\bar{3}m$	784	2.58	4	0.07	0	0.00	0	0.00	1	0.09	3	0.87
189	$P\bar{6}2m$	749	2.46	98	1.70	0	0.00	8	0.45	0	0.00	91	26.30
223	$Pm\bar{3}n$	576	1.89	0	0.00	0	0.00	0	0.00	0	0.00	0	0.00
71	$Immm$	513	1.69	22	0.38	0	0.00	22	1.23	0	0.00	0	0.00
129	$P4/nmm$	509	1.67	1	0.02	0	0.00	1	0.06	0	0.00	0	0.00
140	$I4/mcm$	496	1.63	0	0.00	0	0.00	0	0.00	0	0.00	0	0.00
193	$P6_3/mcm$	456	1.50	10	0.17	0	0.00	10	0.56	0	0.00	0	0.00
12	$C2/m$	445	1.46	12	0.21	0	0.00	12	0.67	0	0.00	0	0.00
123	$P4/mmm$	405	1.33	4	0.07	0	0.00	1	0.06	0	0.00	3	0.87
127	$P4/mbm$	392	1.29	0	0.00	0	0.00	0	0.00	0	0.00	0	0.00
74	$Imma$	356	1.17	6	0.10	0	0.00	6	0.34	0	0.00	0	0.00
204	$Im\bar{3}$	348	1.14	0	0.00	0	0.00	0	0.00	0	0.00	0	0.00
59	$Pmmn$	322	1.06	1	0.02	0	0.00	0	0.00	0	0.00	1	0.29
136	$P4_2/mnm$	320	1.05	0	0.00	0	0.00	0	0.00	0	0.00	0	0.00

Table SI4. Space group distribution within empty perfect kagome entries. The number of entries and the fraction corresponding to the total number of those entries are listed. The highest possible projections into plane groups are determined using Bilbao Crystallographic Server.[23]

Space group	Number	Fraction, %	Plane group projection
227	1379	51.21	*p6mm*
191	938	34.83	*p6mm*
166	181	6.72	*p6mm*
194	93	3.45	*p6mm*
216	79	2.93	*p*31*m*
164	6	0.22	*p6mm*
187	5	0.19	*p*3*m*1
225	5	0.19	*p6mm*
174	3	0.11	*p*3
1	1	0.04	*p*1
156	1	0.04	*p3m1*
160	1	0.04	*p*31*m*
186	1	0.04	*p6mm*

Table SI5. Space group distribution within filled perfect kagome entries. The number of entries and the fraction corresponding to the total number of those entries are listed. The highest possible projections into plane groups are determined using Bilbao Crystallographic Server.[23]

Space group	Number	Fraction, %	Plane group projection
221	937	83.89	*p6mm*
194	68	6.09	*p6mm*
166	49	4.39	*p6mm*
191	35	3.13	*p6mm*
225	12	1.07	*p6mm*
227	6	0.54	*p6mm*
164	3	0.27	*p6mm*
183	2	0.18	*p6mm*
146	2	0.18	*p*3
229	1	0.09	*p6mm*
217	1	0.09	*p*31*m*
187	1	0.09	*p*3*m*1

Table SI6. Space group distribution within empty distorted kagome entries. The number of entries and the fraction corresponding to the total number of those entries are listed. The highest possible projections into plane groups are determined using Bilbao Crystallographic Server.[23]

Space group	Number	Fraction, %	Plane group projection
194	1182	66.29	*p*6*mm*
166	242	13.57	*p*6*mm*
216	182	10.21	*p*31*m*
71	22	1.23	*c*2*mm*
191	17	0.95	*p*6*mm*
141	17	0.95	*p*4*mm*
187	14	0.79	*p*3*m*1
12	12	0.67	*p*2*mm*
63	11	0.62	*c*2*mm*
193	10	0.56	*p*6*mm*
189	8	0.45	*p*31*m*
186	8	0.45	*p*6*mm*
164	7	0.39	*p*6*mm*
74	6	0.34	*c*2*mm*
157	5	0.28	*p*31*m*
139	5	0.28	*p*4*mm*
69	5	0.28	*p*2*mm*
65	5	0.28	*c*2*mm*
1	4	0.22	*p*1
10	3	0.17	*p*2*mm*
227	2	0.11	*p*6*mm*
174	1	0.06	*p*6
160	1	0.06	*p*31*m*
156	1	0.06	*p*3*m*1
148	1	0.06	*p*6
146	1	0.06	*p*3
143	1	0.06	*p*3
129	1	0.06	*p*4*mm*
125	1	0.06	*p*4*mm*
115	1	0.06	*p*4*mm*
72	1	0.06	*c*2*mm*
70	1	0.06	*c*2*mm*
62	1	0.06	*c*2*mm*
38	1	0.06	*p*2*mm*
8	1	0.06	*cm*
6	1	0.06	*pm*

Table SI7. Space group distribution within filled distorted kagome entries. The number of entries and the fraction corresponding to the total number of those entries are listed. The highest possible projections into plane groups are determined using Bilbao Crystallographic Server.[23]

Space group	Number	Fraction, %	Plane group projection
194	195	56.36	*p6mm*
189	91	26.30	*p*31*m*
166	33	9.54	*p6mm*
63	4	1.16	*c2mm*
229	3	0.87	*p6mm*
163	3	0.87	*p6mm*
123	3	0.87	*p4mm*
11	3	0.87	*p2mg*
164	2	0.58	*p6mm*
146	2	0.58	*p*3
160	1	0.29	*p*31*m*
139	1	0.29	*p4mm*
107	1	0.29	*p4mm*
69	1	0.29	*p2mm*
62	1	0.29	*c2mm*
59	1	0.29	*c2mm*
47	1	0.29	*p2mm*

Table SI8. Most common structure types (notation as specified in ICSD outputs) within the kagome database and individual classes of kagomes.

Structure Type	kagome	Empty perfect	Empty distorted	Filled perfect	Filled distorted
Laves(cub)#$MgCu_2$	1376	1376	0	0	0
Auricupride#$AuCu_3$	934	0	0	934	0
Laves(2H)#$MgZn_2$	802	4	798	0	0
$CaCu_5$	644	644	5	0	0
Be_5Au#$SnMgCu_4$	231	65	166	0	0
$PuNi_3$	166	30	136	0	0
$MgFe_6Ge_6$#$HfFe_6Ge_6$	162	162	0	0	0
Ni_3Sn	131	0	0	9	122
Th_2Ni_{17}	130	0	130	0	0
YCo_6Ge_6	111	111	1	0	0

SI3. Details on topological characterisation

Table SI9. Distribution of topological materials properties across different classes of kagome numbers correspond to merged entries). The entries from the kagome database were matched with the ones in the MFD[24] using their ICSD collection numbers. The full names and definitions of the topological properties are provided below.

Topological property	Empty perfect	Empty distorted	Filled perfect	Filled distorted
ESFD	299	141	288	74
Trivial	30	29	2	5
ES	135	201	78	43
SEBR	85	78	30	30
NLC	7	18	0	3

The topological properties of identified materials are defined in the previous study according to the characteristics of the band structure topology near the Fermi level:[24]

Enforced Semimetal (ES): There exist symmetry-enforced band crossings along high-symmetry points and paths in the Brillouin zone while the crossing may not be at the Fermi level.

Enforced Semimetal with Fermi Degeneracy (ESFD): There are symmetry-enforced degenerate states for the bands at the Fermi level at high-symmetry points.

Split Elementary Band Representation (SEBR): The sets of electron bands at high-symmetry points of the Brillouin zone near the Fermi level can be expressed as a linear combination of elementary band representation (EBR), or they can be express as a linear combination of EBR plus disconnected branches of EBR. The band structures of this class resemble that of graphene, whose bands around the Fermi level form one EBR (e.g. p_z orbitals at the atomic sites) when they are gapped by spin-orbit coupling.[24]

No Linear Combination of Elementary Band Representation (NLC): The sets of electron bands at high-symmetry points of the Brillouin zone cannot be expressed as a linear combination of elementary band representations or they cannot be expressed as a linear combination of EBR plus disconnected branches of EBR. This non-linear mixture of two sets of EBRs makes the band structures that may be tuned to trivial or non-trivial topology, depending on the details of gap-opening and closing behaviour of a material through external strains or alloying.[25]

Trivial topology (Trivial): The band structure with a gap that does not exhibit topological features. The band structures can be continuously deformed to those of an atomic insulator without closing the energy gap or breaking a symmetry.

Table SI10. Most common structure types (more than 2 entries, notation as specified in ICSD outputs) with the topological material properties associated with individual classes of kagomes (numbers correspond to merged entries). Ce_5Co_{19} is highlighted as a potential underexplored kagome family with 12 more entries in our database in addition to 2 acknowledged in MFD.[24]

Space group	Structure Type	ESFD	Trivial	ES	SEBR	NLC
		Empty perfect				
**227**		**159**	**21**	**35**	**46**	**0**
	Laves(cub)#$MgCu_2$	158	21	35	46	0
**191**		**99**	**0**	**60**	**17**	**0**
	$CaCu_5$	62	0	45	13	0
	$MgFe_6Ge_6$#$HfFe_6Ge_6$	34	0	15	4	0
	Zr_4Al_3	3	0	0	0	0
**166**		**17**	**6**	**16**	**17**	**0**
	$PuNi_3$	5	1	2	2	0
	Gd_2Co_7	0	3	12	8	0
	W_6Fe_7	6	0	1	3	0
	Y_2Rh_3Ge	3	0	0	0	0
	Ce_5Co_{19}	***1***	***0***	***0***	***1***	***0***
**194**		**0**	**0**	**19**	**5**	**0**
	Laves(4H)#$MgNi_2$	0	0	17	5	0
**216**		**22**	**3**	**4**	**0**	**7**
	Be_5Au#$SnMgCu_4$	22	3	4	0	7
		Empty distorted				
**194**		**28**	**9**	**180**	**44**	**0**
	Laves(2H)#$MgZn_2$	6	9	125	32	0
	Th_2Ni_{17}	9	0	14	2	0
	Laves(4H)#$MgNi_2$	0	0	17	3	0
	$TbCu_9Mg_2$#$CeNi_3$	12	0	12	3	0
	Ce_2Ni_7	0	0	9	2	0
	$CeNi_5Sn$	0	0	3	2	0
**166**		**39**	**3**	**8**	**25**	**0**
	$PuNi_3$	28	1	0	15	0
	W_6Fe_7	2	1	1	2	0
	Th_2Zn_{17}	4	0	6	3	0
**216**		**57**	**12**	**3**	**0**	**10**
	Be_5Au#$SnMgCu_4$	57	12	3	0	10
**71**		**7**	**1**	**0**	**4**	**0**
	$ScFe_6Ga_6$	5	0	0	4	0
	$HoFe_6Sn_6$	2	1	0	0	0
		Filled perfect				
**221**		**260**	**1**	**52**	**21**	**0**
	Auricupride#$AuCu_3$	260	1	52	21	0
**194**		**6**	**0**	**20**	**3**	**0**
	Ni_3Sn	3	0	4	0	0
	$TiNi_3$	0	0	13	3	0
**166**		**7**	**1**	**2**	**6**	**0**
	$BaPb_3$	5	0	2	5	0
**191**		**9**	**0**	**3**	**0**	**0**
	CoSn	9	0	3	0	0
**225**		**5**	**0**	**1**	**0**	**0**
	Ca_7Ge	4	0	1	0	0

Space group	Structure Type	ESFD	Trivial	ES	SEBR	NLC
		Filled distorted				
**194**		**39**	**4**	**36**	**8**	**39**
	Ni_3Sn	37	3	19	5	0
	$TiNi_3$	0	0	13	2	0
**189**		**26**	**0**	**5**	**16**	**26**
	ZrNiAl#Fe_2P	26	0	5	16	0
**166**		**3**	**1**	**1**	**6**	**3**
	$BaPb_3$	1	0	0	2	0
	$HoAl_3$	2	0	0	1	0

SI4. Detailed Methods of ML Classification and Chemical Similarity Ranking

4.1. Binary Classifiers

Phases were aggregated into phase fields and assigned to the positive class if any phase within them contained kagome layers. Since only quaternary phase fields were investigated, any phase fields that did not contain four elements were discarded. All phase fields were permuted to mitigate the impact of vector format on model performance, giving a dataset of 25848 permuted quaternary phase fields (1077 unique phase fields). Phase fields were featurised with a library of 28 atomic Magpie features, giving vectors of length 112 for each phase field.

Eight different ML classification models were built with Scikit learn (version 1.3.2): five variations of decision tree algorithms (basic, boosting, bagging, random forest and gradient boosting), k-nearest neighbours and two linear support vector machines with and without a stochastic gradient descent (SGD) algorithm. The phase fields were split into training, validation and testing sets in a ratio of 81:9:10, ensuring all permutations of the same phase field were present in the same set. Grid searches were conducted to optimise the hyperparameters of the models where applicable. The models were trained on the 81% training set and evaluated on the 9% validation set with five metrics: accuracy, precision, recall, F1 and area under the receiver operator curve (AUC), whilst the respective hyperparameters of each model were varied. The five metrics were plotted against the varying hyperparameters, and the optimum values were then qualitatively chosen based on where the metrics plateaued. At the plateau, changing the hyperparameter no longer improves model performance, which is why the hyperparameters were selected at this point. Once the grid searches were complete, the training and validation sets were combined to form a new 90% training set. The final performance of each model was then determined on the withheld 10% testing using a separate metric, the Matthew's correlation coefficient (MCC), with the boosted decision tree achieving the highest score of 0.74.

The boosted decision tree architecture consists of an ensemble of decision trees that are trained sequentially. During training, the training entries that are misclassified by a single decision tree are weighted higher in the training of the following tree, so that there is a higher reward for correcting previously incorrect predictions. The aim of each individual decision tree in the ensemble is to interrogate and subsequently separate the training entries into multiple groups, where each group consists of only one class. In reality, training entries may not be cleanly separated by a decision tree, so a class probability can be calculated from the proportions of each class in a group. When a trained decision tree is used to classify an unseen query entry, the entry is interrogated in the same way and falls into one of the groups. The entry will then be assigned to a class, dependent on whether the class probability of the group meets a predetermined threshold. Boosted decision trees calculate the probability of an entry belonging to a class in each sequential decision tree and then produce a weighted average over all trees, where the weights are related to the calculated accuracy of each individual tree during training. The kagome probability used to rank a query phase field was defined as the probability of its proposed classification, as explained above.

4.2. Chemical Similarity Ranking

Phase fields containing metals (as defined according to the criteria explained in SI1.1) with an atomic number greater than 87 were removed from the dataset when studying chemical feasibility, giving a ground truth dataset of 24936 permuted quaternary phase fields (1039 unique phase fields). A query dataset of 454087 potentially unexplored phase fields was constructed from every quaternary combination of elements within the ground truth dataset, provided that the combination was not already in the ground truth dataset. The same Magpie libraries were used to featurise the phase fields,

however an undercomplete autoencoder (TensorFlow version 2.15.0) was employed to reduce the dimensionality of the atomic MagPie vectors. The first autoencoder had the architecture [***input, normalisation, hidden, output, reshape***], all with sizes equal to the input data except the hidden layer, the size of which was the specified number of latent features. Interior layers were densely connected. The autoencoder was trained to minimise the mean squared error between the inputted Magpie vectors and the reconstructed output. The atomic latent vectors were extracted from the optimised model as the compression emphasises the most relevant features and minimises redundant information. The Magpie vectors were compressed seven times to give meaningful features of between 2 and 8 dimensions.

A second, overcomplete, autoencoder (TensorFlow version 2.15.0) was then used to assess the chemical similarity, and by proxy, chemical feasibility of the query dataset. The second autoencoder had densely connected layers structured [***input, normalisation,*** $\boldsymbol{hidden_1}$, $\boldsymbol{hidden_2}$, $\boldsymbol{hidden_3}$, ***output***], with the $\mathbf{hidden_2}$ layer having 120 nodes and the other layers being of equal size to the input vectors. The model was trained and tested eight times, using each of the compressed sets of feature vectors (between 2 and 8 dimensions) and uncompressed Magpie features to represent the ground truth and query datasets. The model was trained on the ground truth phase fields containing reported structures. A query phase field that is then able to be represented in and reconstructed from the same latent space as reported phase fields must be similar in feature space, and hence also chemically. A lower reconstruction error, given by the root sum of squared errors, is therefore indicative of chemical similarity.

4.3. Candidate Selection

The maximum fractional difference (MFD) between the proportion of ground truth and query phase fields below a given reconstruction error was used to select the threshold at which to assign query phase fields to the feasible class. A larger MFD implies a more conservative model, which is preferred, so the ranking of reconstruction errors that produced the largest MFD and the corresponding threshold were chosen. Permutations within the dataset were then averaged and compared to the threshold. The MFD was also calculated using the mean reconstruction errors to assess the impact, if any, on the threshold. Phase fields with a reconstruction error below the determined threshold were assigned to the chemically feasible class, with the reconstruction error being a measure of how similar the query phase fields were to reported phase fields.

The chemically feasible query phase fields were then inputted into the best performing binary classifier model, which was found to be the decision tree employing a boosting algorithm. Each phase field was then characterised by two metrics: reconstruction error outputted from the VAE, which assesses chemical feasibility, and the probability of containing at least one compound with kagome layers, calculated by the decision tree model. Pareto fronts were plotted to optimise both metrics simultaneously.

Table SI11. Scores achieved by each of the binary classifier models on the withheld 10% testing set. Model hyperparameters were selected by conducting grid searches (see Section 4.1). The final performances of the models were evaluated on the MCC, which was not used in the optimisation of the hyperparameters. SVM models are inherently deterministic and do not use distributions to assign datapoints to a class. Hence, AUC cannot be used as a metric because it evaluates model performance at different probability thresholds.

**Method**	**Accuracy**	**Precision**	**Recall**	**F1**	**AUC**	**MCC**
Simple Tree	0.888	0.694	0.647	0.670	0.815	0.603
Boosting	0.929	0.895	0.675	0.770	0.936	0.739
Bagging	0.927	0.852	0.706	0.772	0.933	0.734
Random Forest	0.926	0.861	0.693	0.768	0.931	0.731
Gradient Boosting Tree	0.870	1.00	0.261	0.414	0.887	0.475
K-Neighbours	0.880	0.667	0.632	0.649	0.777	0.576
SVM SGD	0.852	0.901	0.180	0.300	nan	0.363
SVM Linear	0.850	0.784	0.200	0.318	nan	0.346

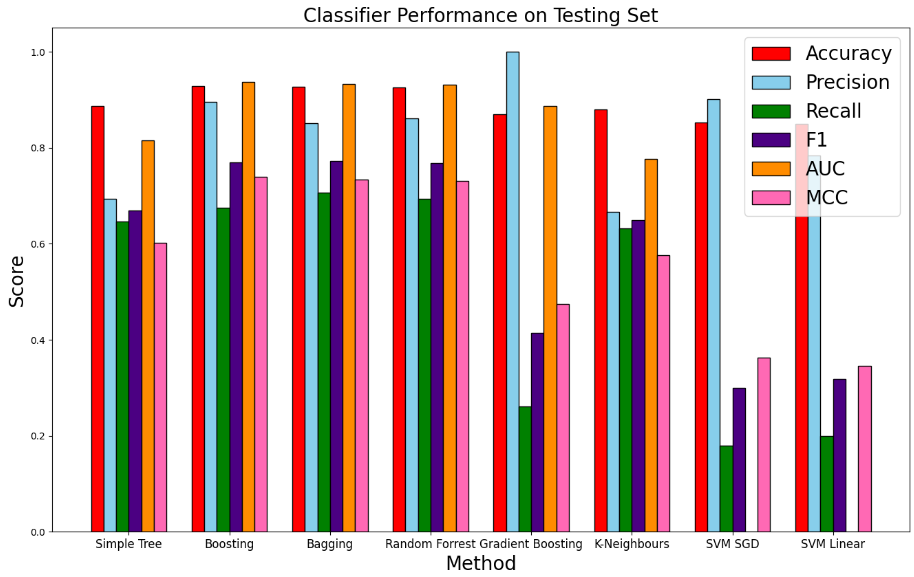

Figure SI8. Plots of accuracy (red), precision (blue), recall (green), F1 (purple), AUC (orange), MCC (pink) that each model achieved on the withheld 10% testing set. MCC and F1 values tend to be lower as they are more reliable metrics. Accuracy and AUC can be skewed by imbalances in the dataset, whilst precision and recall measure the conservativeness and leniency of a model but are not meaningful independently.

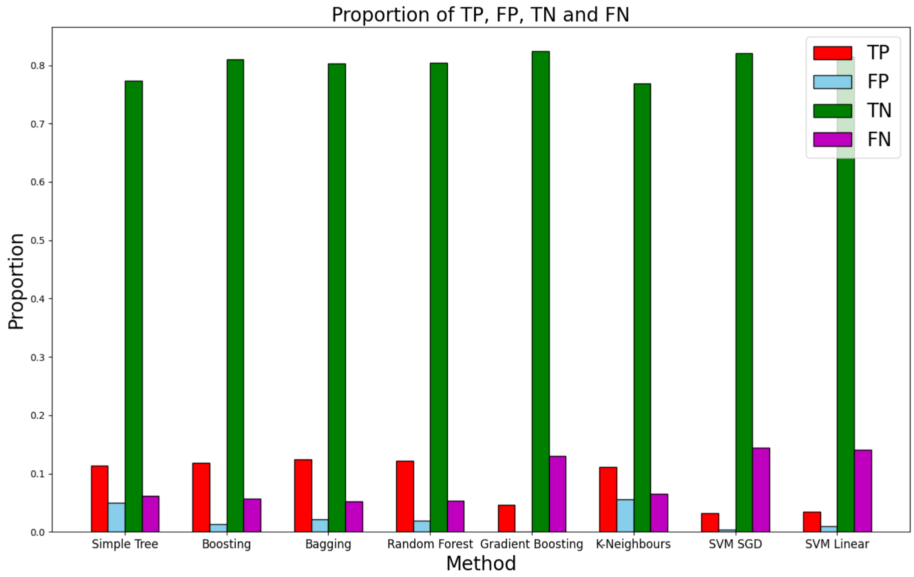

Figure SI9. Proportion of true positive (red), false positive (blue), true negative (green) and false negative (purple) assignments made by each classifier on the withheld 10% testing set. The large proportion of true negative assignments emphasises the class imbalances. A perfect model would have only true positive and true negative assignments. Notably, the gradient boosting decision tree made only correct positive predictions.

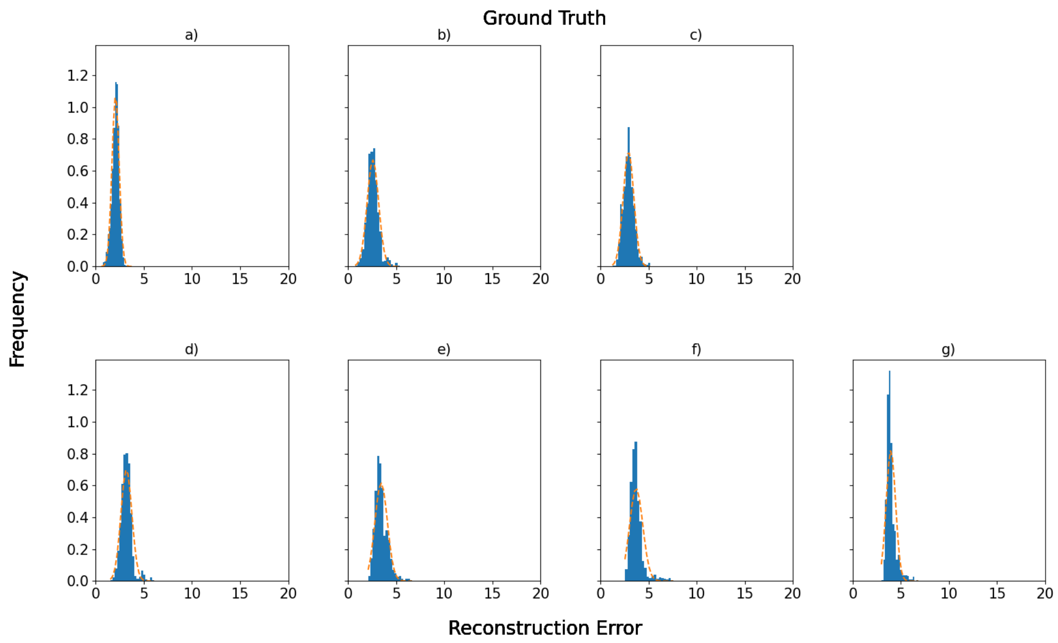

Figure SI10. Histograms of the reconstruction errors between the inputted and reconstructed ground truth phase fields, after input into an overcomplete autoencoder. The autoencoders were trained on the ground truth dataset, with the reconstruction errors being taken from a separate run with the trained model. Plots a-g show the variation in reconstruction errors when the dataset was represented by the compressed Magpie vectors of between 2 and 8 dimensions respectively.

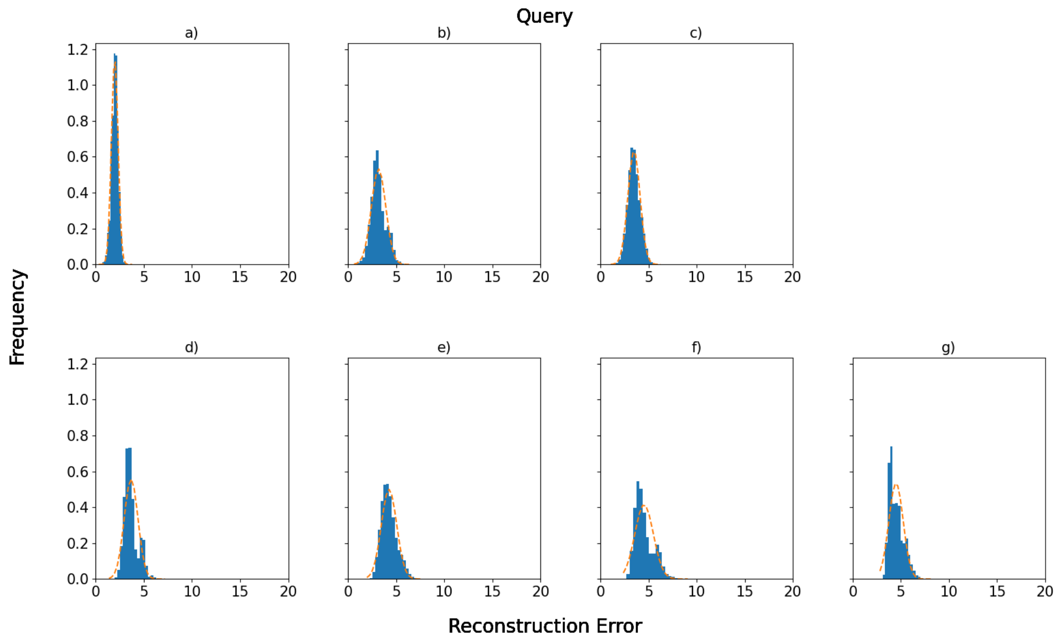

Figure SI11. Histograms of the reconstruction errors between the inputted and reconstructed query phase fields, after input into an overcomplete autoencoder. Since the autoencoder model was already trained on the ground truth dataset, query phase fields that achieve lower reconstruction errors were represented in and reconstructed well from the same latent space. Reconstruction error is therefore an indicator of chemical similarity of query phase fields to reported, stable ground truth phase fields. Plots a-g show the variation in reconstruction errors when the dataset was represented by the compressed Magpie vectors of between 2 and 8 dimensions respectively.

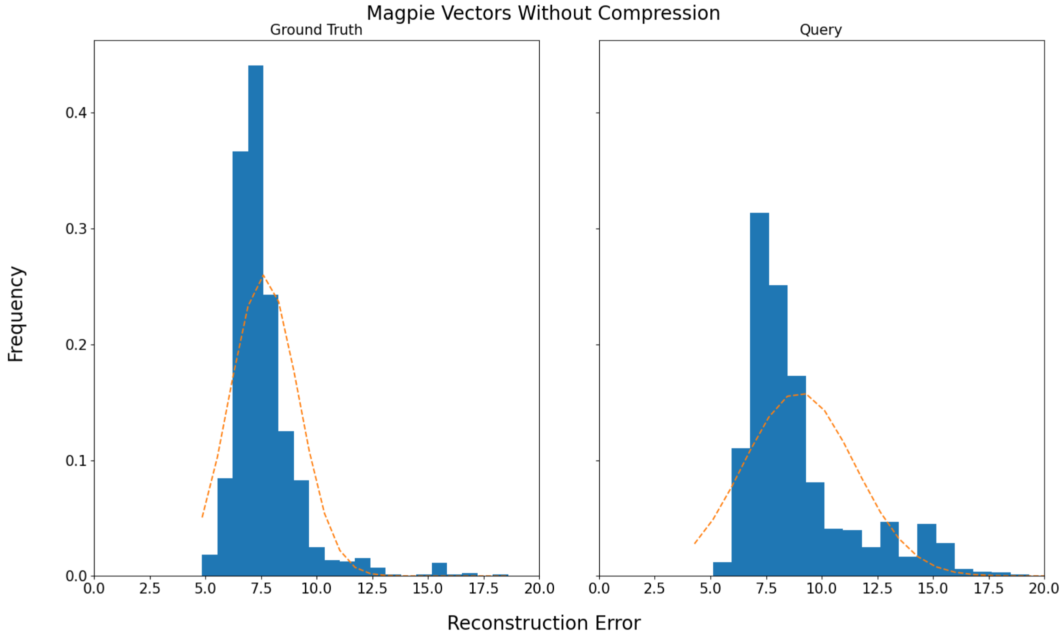

Figure SI12. Histograms of reconstruction errors between the inputted and reconstructed phase fields of the ground truth (left) and query (right) datasets. For both datasets, the reconstruction errors were generally higher, demonstrating how compressing the ‘off-the-shelf’ Magpie vectors emphasises the more meaningful features.

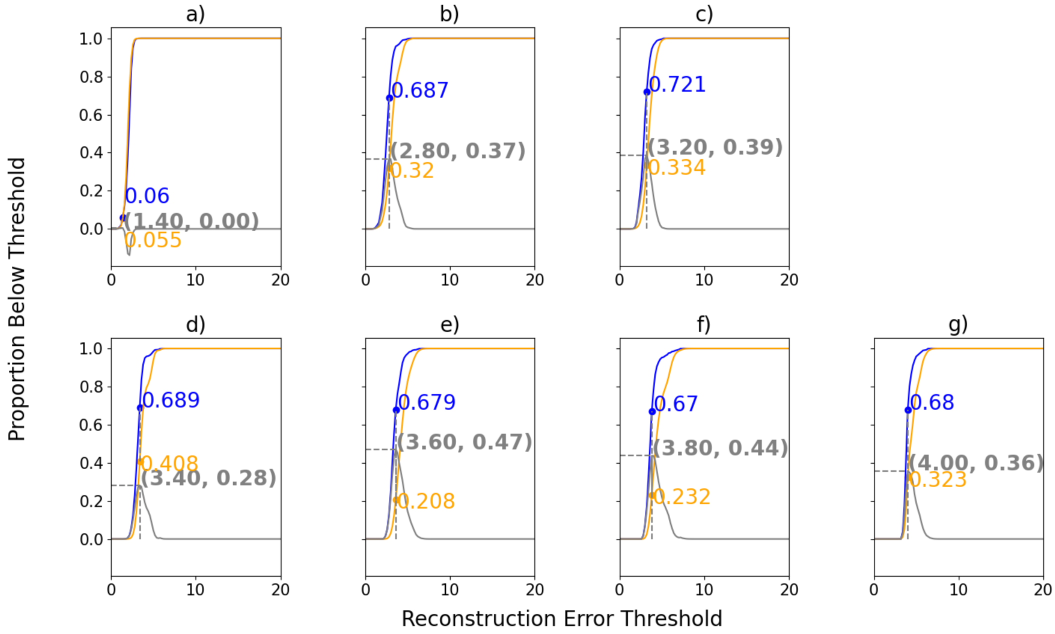

Figure SI13. Fraction of the ground truth (blue) and query (orange) phase fields below a given reconstruction error, with the fractional difference (grey) between them. The maximum fractional difference (MFD) and corresponding proportion at which it occurs are labelled. A larger MFD generally indicates a more conservative model; the associated reconstruction error can then be used as a threshold to assign query phase fields to the chemically feasible class. Plots a-g show the MFD calculated for each pair of rankings, achieved using Magpie vectors compressed to between 2 and 8 dimensions.

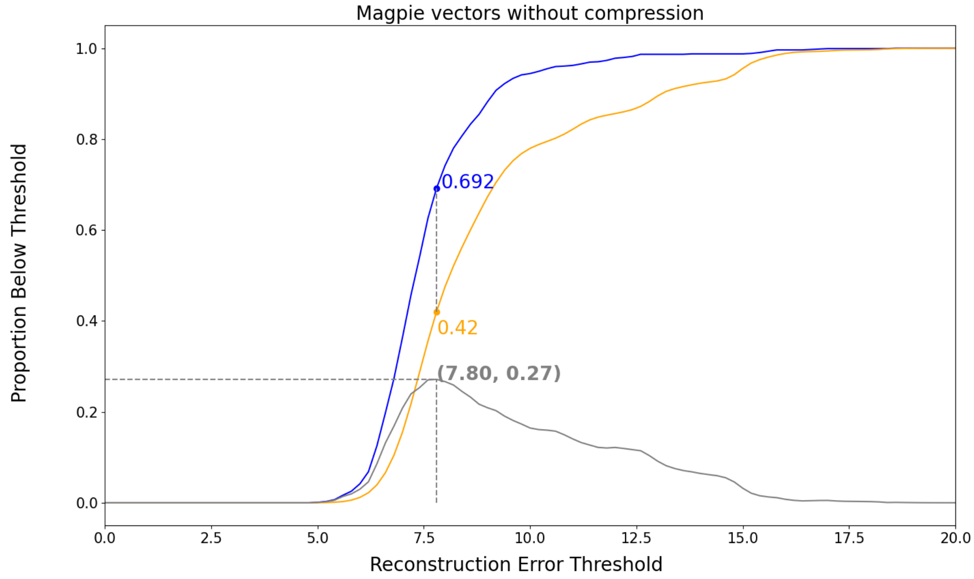

Figure SI14. Fraction of the ground truth (blue) and query (orange) phase fields below a given reconstruction error with the fractional difference (grey) between them, plotted from the ranking that employed “off-the-shelf” Magpie vectors. Although both datasets achieved higher reconstruction errors, the differentiation between the two is more significant when assigning query datapoints to a class of positive datapoints. The MFD could still be used from a ranking that gave large reconstruction errors, provided the query dataset reconstruction errors were larger than that of the ground truth dataset.

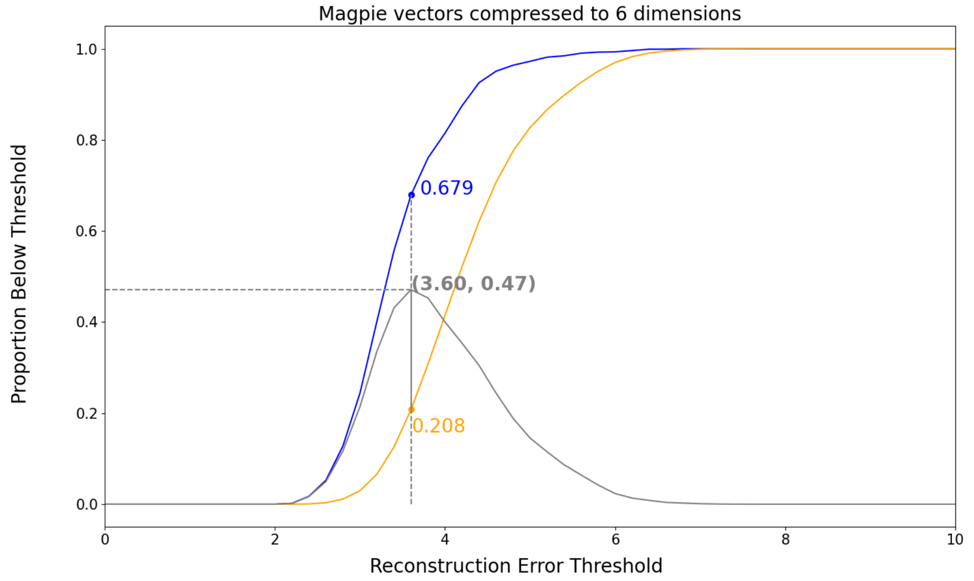

Figure SI15. Enhanced plot of the MFD from the ranking that used Magpie vectors compressed to 6 dimensions, which gave the largest MFD value of 0.47 at a threshold of 3.60. A larger MFD generally results in a more conservative assignment because the maximum number of phase fields that can be below the threshold is *1 – MFD value*. In practice, the proportion assigned to the positive class tends to be much lower. For example, this threshold assigned 20.7% of query phase fields to the chemically feasible class, despite the theoretical maximum being 53%.

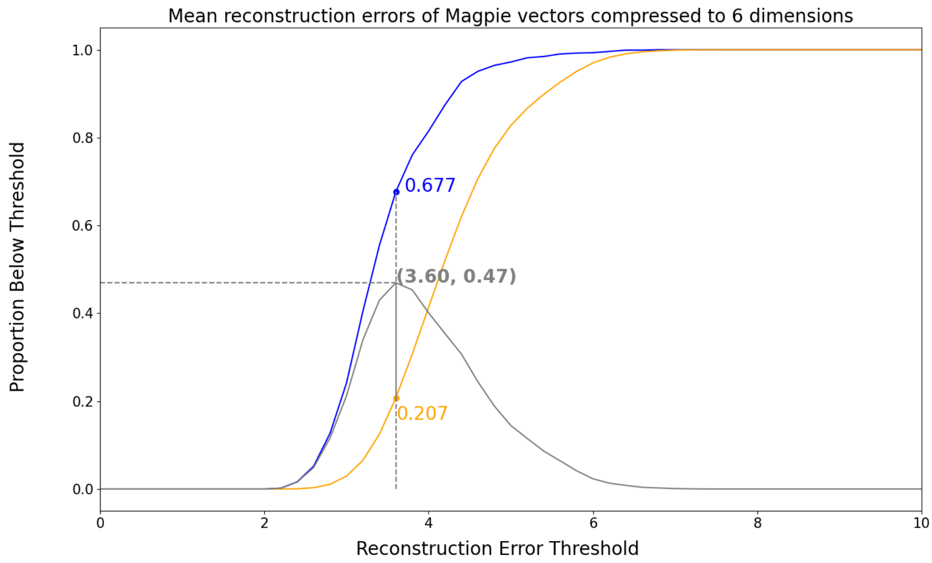

Figure SI16. MFD plot of mean phase field reconstruction errors from the ranking that used Magpie vectors compressed to 6 dimensions. It was possible that some phase fields may have had permutations on either side of the threshold. The plot was constructed using the mean reconstruction errors to ensure that the distributions did not change significantly, which is evidenced by the identical MFD, threshold and proportion of the datasets below the threshold.

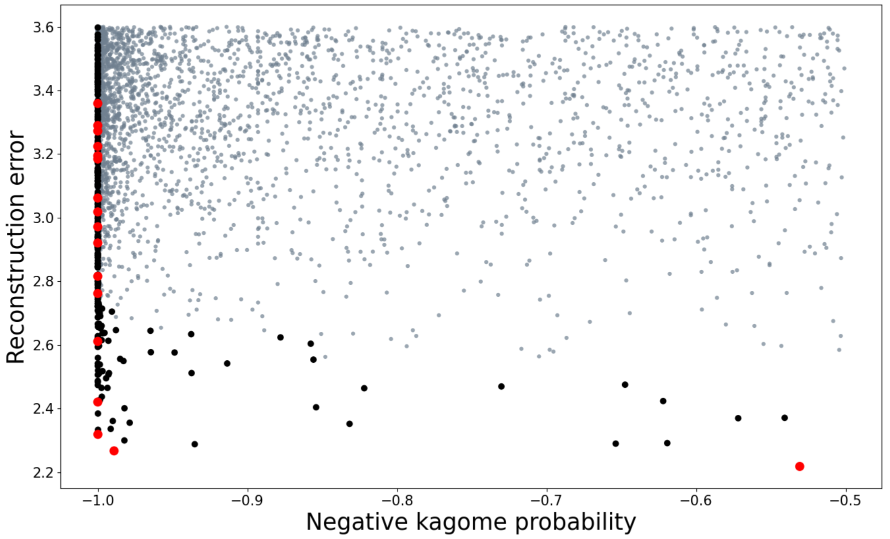

Figure SI17. The probability that each phase field contained at least one phase with kagome layers, and the reconstruction errors achieved by the autoencoder were optimised simultaneously by plotting Pareto fronts. To transform the problem into a minimisation problem in both dimensions, the probability was multiplied by −1 giving the negative kagome probability on the horizontal axis. Phase fields with a lower reconstruction error and more negative kagome probability would be the most ideal candidates, indicated by the low numbered front that they lie on. 17 phase fields were found to lie on the first front, shown in red. Phase fields on fronts two to ten are coloured black and all other phase fields are coloured grey.

Table SI12. Phase fields on the top 5 Pareto fronts based on the probability that they contain at least one phase with kagome layers and their reconstruction errors achieved by the autoencoder. Quaternary phases reported to contain kagome layers in Persons' database (Hits in PCD)[26] are listed.

Ranking	Phase Field	RE	Kagome Probability	Front	Hits in PCD	Ranking	Phase Field	RE	Kagome Probability	Front	Hits in PCD
1	Mg Ni Pr Y	3.36	1	1	0	57	Co La Nd Ni	3.51	1	4	0
2	Co Gd La Ni	3.29	1	1	0	58	Cr La Ni Pr	3.48	1	4	0
3	Ce La Mg Ni	3.27	1	1	$MgLa_{0.5}Ce_{0.5}Ni_4$[27]	59	Ce Er Mn Ni	3.32	1	4	0
4	Co La Ni Y	3.22	1	1	0	60	Ce Mn Ni Sm	3.31	1	4	0
5	Al Mo Ni Pr	3.19	1	1	0	61	Ce Cr La Ni	3.3	1	4	$Ce_{0.4}Cr_2La_{0.6}Ni_3$[28]
6	Ca Ni Sc Y	3.18	1	1	0	62	Mg Ni Tb Y	3.23	1	4	0
7	Cr La Li Ni	3.06	1	1	0	63	Al Mg Ni Pr	3.11	1	4	0
8	Co Ho Mg Ni	3.02	1	1	0	64	Cu La Mn Ni	3.01	1	4	0
9	Gd Li Mg Ni	2.97	1	1	0	65	La Mg Mo Ni	2.94	1	4	0
10	Gd Mg Ni Sc	2.92	1	1	0	66	Ni Sc Y Zr	2.94	1	4	0
11	Hf Nb Ni Ti	2.82	1	1	0	67	Mg Mo Ni W	2.91	1	4	0
12	La Mg Mn Ni	2.76	1	1	$MgLaMn_{0.3}Ni_{3.7}$,[29] $MgLaMn_{0.5}Ni_{3.5}$, $MgLaMnNi_3$[30]	68	Al Ce Cr Ni	2.9	1	4	0
13	Ga Mn Sc Sn	2.61	1	1	0	69	Hf Mn Nb Ni	2.82	1	4	0
14	Nb Ni Sc Ti	2.42	1	1	0	70	Ge Mn Sn Zr	2.75	1	4	0
15	Mn Ni Ti Zr	2.32	1	1	$Zr_{0.05}Ti_{0.95}Mn_{1.5}Ni_{0.5}$,[31] $Zr_{0.05}Ti_{0.95}Mn_{1.5}Ni_{0.5}$,[32] $Zr_{0.8}Ti_{0.2}Mn_{0.7}Ni_{1.3}$[33]	71	Gd Mg Ni Ti	2.73	1	4	0
16	Nb Ni Ti V	2.27	0.989	1	0	72	Mg Mn Ti Zr	2.62	1	4	0
17	Cr Ni Ti V	2.22	0.531	1	0	73	Cr Nb Ni Zr	2.56	1	4	0
18	La Mg Ni Pr	3.45	1	2	$La_{0.5}Pr_{0.5}MgNi_4$[34]	74	Co Mn Ni Zr	2.52	1	4	0
19	Co La Ni Tb	3.42	1	2	0	75	Mn Sc Ti V	2.51	0.999	4	0
20	Ce Mn Ni Tb	3.3	1	2	0	76	Ag Al Ga Ni	2.47	0.998	4	0
21	Ce Dy Mn Ni	3.28	1	2	0	77	Cr Ni Sc V	2.44	0.997	4	0
22	Co Gd Ni Y	3.19	1	2	0	78	Ni Ti V Zr	2.36	0.99	4	$Ni_{1.412}Ti_{0.176}V_{0.706}Zr_{0.706}$, $Ni_{1.412}Ti_{0.353}V_{0.706}Zr_{0.529}$,[35] $Ni_{1.5}Ti_{0.5}V_{0.5}Zr_{0.5}$,[36] $Ni_{1.3}Ti_{0.5}V_{0.7}Zr_{0.5}$,[33] $Ni_{1.25}Ti_{0.5}V_{0.75}Zr_{0.5}$[37]
23	Ce Cr Cu Ni	3.12	1	2	0	79	Mg Nb Ni Ti	2.36	0.979	4	0
24	Hf Nb Ni Zr	3.02	1	2	0	80	Ga Ge In Mn	2.35	0.832	4	0
25	Co Dy Mg Ni	3.01	1	2	0	81	Mn Ni Sc Ti	2.29	0.619	4	0
26	Cr La Mg Ni	2.85	1	2	$La_2Cr_{0.05}Mg_{16.42}Ni_{0.53}$[38]	82	Co La Ni Pr	3.53	1	5	0

27	Gd Ni Sc Ti	2.8	1	2	0	83	Ce Cr Ni Pr	3.51	1	5	0
28	Cr Mn Sc Zr	2.76	1	2	0	84	Co Gd Ni Pr	3.5	1	5	0
29	Co Mg Ni Sc	2.62	1	2	0	85	Gd Mg Ni Pr	3.42	1	5	0
30	Ge Mn Sn Ti	2.53	1	2	0	86	Ce La Ni Sc	3.37	1	5	0
31	In Mn Sn Ti	2.49	1	2	0	87	Gd Mg Ni Sm	3.31	1	5	0
32	Mn Nb Ni Zr	2.47	1	2	0	88	Co La Lu Ni	3.25	1	5	0
33	Mn Ni V Zr	2.38	1	2	$Mn_{0.5}Ni_{1.3}V_{0.2}Zr$,[33] $Mn_{0.5}NiV_{0.5}Zr$[39]	89	Ce Co Ni Y	3.25	1	5	0
34	Ni Sc Ti V	2.33	1	2	0	90	Co Nd Ni Zr	3.24	1	5	0
35	Ga Ge Mn Ti	2.3	0.982	2	0	91	Er Ge Mn Sn	3.18	1	5	$ErGe_2Mn_6Sn_4$[40]
36	Mn Nb Ni V	2.29	0.935	2	0	92	Co Mg Sc Y	3.13	1	5	0
37	Co La Ni Sm	3.43	1	3	0	93	Ce Mn Ni Y	3.11	1	5	0
38	Ce Ho Mn Ni	3.29	1	3	0	94	Ga Ho Mn Sn	3.03	1	5	$HoMn_6GaSn_5$[41]
39	Ce La Ni V	3.24	1	3	0	95	Co Cu Mn Zr	3.02	1	5	0
40	Cr Er Ge Mn	3.1	1	3	0	96	Ce Mg Ni Sc	2.99	1	5	0
41	Co Mg Ni Sm	3.05	1	3	0	97	Co Er Mn Ni	2.98	1	5	0
42	Co Gd Mg Ni	2.91	1	3	0	98	La Mg Ni Sc	2.96	1	5	0
43	Hf Ni Sc V	2.91	1	3	0	99	Al Gd Mo Ni	2.92	1	5	0
44	Hf Ni Sc Ti	2.86	1	3	0	100	Al Ni Sc Y	2.85	1	5	0
45	Mo Ni Ti W	2.81	1	3	0	101	Ni Sc V Y	2.78	1	5	0
46	La Mg Ni Ti	2.77	1	3	0	102	Hf Mn Ni V	2.74	1	5	0
47	Cr Ni Sc Zr	2.61	1	3	0	103	Ce Mn Ni Ti	2.71	1	5	0
48	Al Co Mg Ni	2.59	1	3	0	104	Cr Mn V Zr	2.63	1	5	0
49	Ge Mn Sb Ti	2.54	1	3	0	105	Cr Ge Mn Sb	2.62	1	5	0
50	Ni Sc Ti Zr	2.51	1	3	0	106	Cr Mn Sc Ti	2.54	1	5	0
51	Nb Ni Sc V	2.48	1	3	0	107	Mg Ni V Zr	2.52	0.997	5	0
52	Co Mg Ni Ti	2.43	0.999	3	0	108	Mg Ni Sc V	2.5	0.994	5	0
53	Cr Mn Nb Ni	2.34	0.991	3	0	109	Cr Ni V Zr	2.47	0.994	5	$Zr_{0.75}V_{0.975}Cr_{0.15}Ni_{1.125}$[42]
54	Mg Ni Ti V	2.29	0.654	3	0	110	Cr Ni Ti Zr	2.4	0.982	5	$Zr_{0.5}Ti_{0.5}CrNi$, $Zr_{0.75}Ti_{0.25}Cr_{1.5}Ni_{0.5}$[43]
55	Ce Nb Ni Pr	3.53	1	4	0	111	Ag Ga In Ni	2.37	0.572	5	0
56	Ce La Na Ni	3.52	1	4	0						

SI5. Band structure calculations for $ErMn_6Sn_4Ge_2$

To further highlight the relevance of the predicted phase fields, we carried out an extensively search in the Pearsons crystal database among the top-5 Pareto front quaternary phase fields, and we found that $ErMn_6Sn_4Ge_2$[40] is the only ordered structure that can be modelled with first-principles calculations without complications. This compound originates from the $MgFe_6Ge_6$ structure type (*P*6/*mmm* space group), as shown in Figure S18.[40] Mn atoms form the kagome layer. While a previous experimental study has shown that $ErMn_6Sn_4Ge_2$ exhibits a high Néel temperature at 358 K and an interesting ferrimagnetic transition around 140 K,[40] the electronic structures of this compound is not understood. Therefore, density functional theory calculations with Hubbard *U* corrections (DFT+U) were carried out with the VASP package to exemplify the potential contributions to its low-temperature magnetic states. [44–47]

The ground-state structure was optimized using the PBE+U functional with the spin-orbit coupling (SOC).[44,45] The kinetic energy cutoff is 500 eV for the plane-wave basis set, and a k-point grid of R_k = 40 was used for energy integration in the Brillouin zone (BZ). The total energy convergence criteria are set to 10^{-6} eV for self-consistent steps. And the ionic optimization was converged with the residual forces on each ion lower than 0.01 eV/Å. After structural optimization, the SOC band structures were calculated by considering the Hubbard correction with *U* = 8 eV for Er, as suggested for metallic Er.[46–49]

The calculated magnetic ordering of $ErMn_6Sn_4Ge_2$ is consistent with the ferrimagnetic state observed by Venturini,[40] where the magnetic moments for Mn and Er align in opposite directions. The two magnetic configurations are shown in Figure SI18, where magnetic moments are aligned along the *c*-axis and within the *ab* plane, respectively. The magnetizations of the Mn kagome sublattice sum to around 13 μ_B in good agreement with the experiments. The calculated magnetic moments of Er are around 3 μ_B, mainly contributed by the *f* electrons.[47] The orbital contribution to the magnetic moment of Er is expected to be around 6 μ_B, approximated by Hund's rule.[48] The DFT total energy of the *c*-axis magnetic state is about 7.6 meV/atom higher than that of the *ab* plane alignment, suggesting easy magnetization direction within the *ab* plane.

The band structures and density of states (DOS) of the two magnetic configurations are shown in Figure SI19 and Figure SI20, respectively, where the Mn *d* orbitals contribute to the electronic states near the Fermi level. Notably, Dirac dispersions are observed at the high-symmetry K point, around 0.1 eV above and below the Fermi level (Figure SI19-SI20). These Dirac dispersions are originated from the d_{z^2}, $d_{x^2-y^2}$ and d_{xy} orbitals of the Mn atoms that form the kagome layers (Figure SI19b and c). A flat band is observed near 0.5 eV above the Fermi level with the same orbital parentage. The Dirac-like dispersions near the Fermi level at the A symmetry point are mainly contributed by the d_{z^2}.

While the DOSs of the two magnetic states do not differ significantly, the changes of the Dirac band dispersions around the high-symmetry K point may influence the electron transport of these two states. For the magnetic states along *c*-axis, the Dirac dispersions are linear as shown in Figure SI21a. However, the change of magnetic alignment disrupts that dispersion with wider SOC gaps (Figure SI21b). The *ab*-plane magnetization exhibits a larger SOC gap, turning the linear dispersions at K point into parabolic. Therefore, the stabilization of magnetization along the c-axis holds significant potential for spintronic applications requiring high electron mobilities. The above observations suggest that the ordered kagome 1-6-4-2 compounds such as $ErMn_6Sn_4Ge_2$ can offer promising opportunities to tune magnetic anisotropy by stabilizing ferrimagnetic order along the stacking direction of the kagome layers.

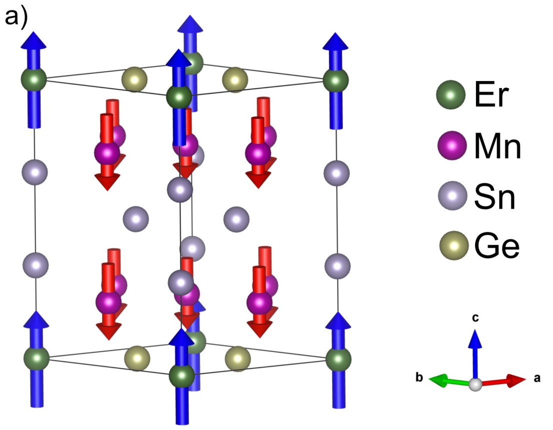

Figure SI18. (a) Ferrimagnetic state of $ErMn_6Sn_4Ge_2$ with magnetic moments of Er and Mn aligns along the *c* axis perpendicular to the kagome layers. (b) Ferrimagnetic state of $ErMn_6Sn_4Ge_2$ with magnetic moments of Er and Mn aligns within the *ab* plane of the unit cell.

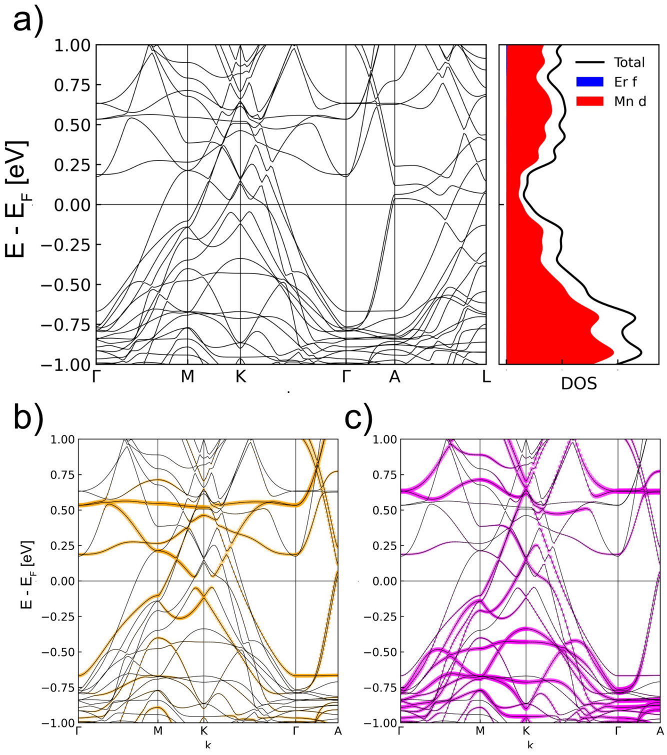

Figure SI19. Band structures of the ferrimagnetic state of $ErMn_6Sn_4Ge_2$ with magnetic moments align along *c*-axis. (a) SOC band structure and DOS with orbital projections from f orbitals of Er and d orbitals of Mn. (b) SOC band structure with orbital projections from d_{z^2} orbitals of Mn. (c) SOC band structure with orbital projections from $d_{x^2-y^2}$ and d_{xy} orbitals of Mn.

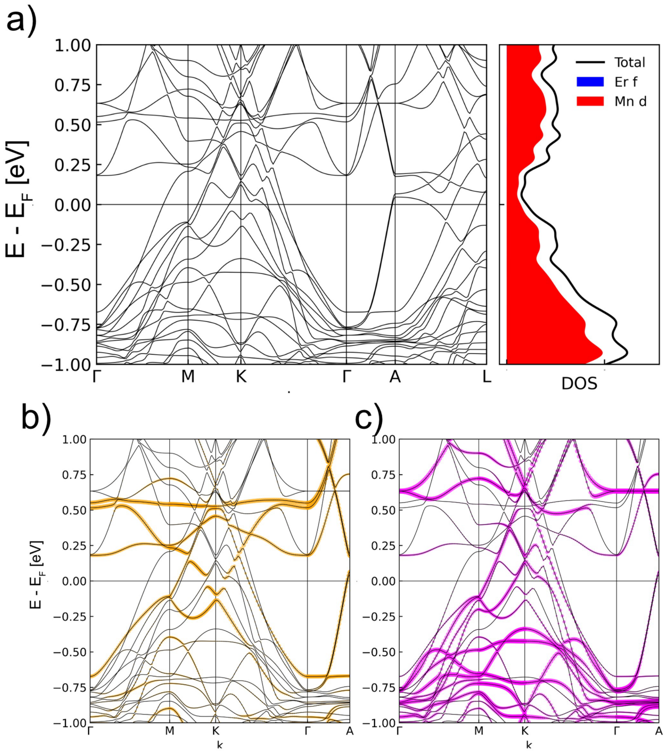

Figure SI20. Band structures of the ferrimagnetic state of $ErMn_6Sn_4Ge_2$ with magnetic moments align within *ab* plane. (a) SOC band structure and DOS with orbital projections from f orbitals of Er and d orbitals of Mn. (b) SOC band structure with orbital projections from d_{z^2} orbitals of Mn. (c) SOC band structure with orbital projections from $d_{x^2-y^2}$ and d_{xy} orbitals of Mn.

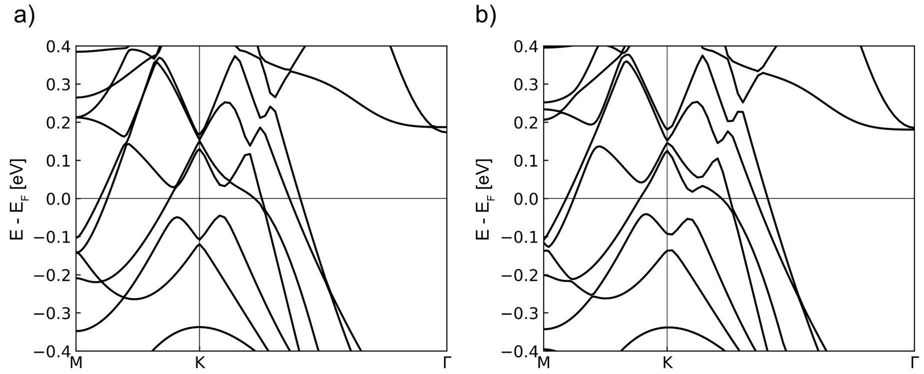

Figure SI21. (a) Dirac band dispersions of the ferrimagnetic state of $ErMn_6Sn_4Ge_2$ with magnetic moments align along *c*-axis. (b) Dirac band dispersions of the ferrimagnetic state of $ErMn_6Sn_4Ge_2$ with magnetic moments align within *ab* plane.

References:

[1] Inorganic Crystal Structure Database - ICSD, FIZ Karlsruhe GmbH, **2022**.

[2] W. Steurer, J. Dshemuchadse, *Intermetallics: Structures, Properties, and Statistics*., Oxford University Press **2016**.

[3] P. M. Neves, J. P. Wakefield, S. Fang, H. Nguyen, L. Ye, J. G. Checkelsky, Crystal Net Catalog of Model Flat Band Materials. *Npj Comput. Mater.* **2024**, *10*, 1.

[4] V. A. Blatov, A. P. Shevchenko, D. M. Proserpio, Applied Topological Analysis of Crystal Structures with the Program Package Topospro. *Cryst. Growth Des.* **2014**, *14*, 3576.

[5] V. A. Blatov, D. M. Proserpio, *ToposPro. Practical Manual. Version 5.0.*, **2020**.

[6] V. A. Blatov, Voronoi–Dirichlet Polyhedra in Crystal Chemistry: Theory and Applications. *Crystallogr. Rev.* **2004**, *10*, 249.

[7] V. A. Blatov, G. D. Ilyushin, O. A. Blatova, N. A. Anurova, A. K. Ivanov-Schits, L. N. Dem'yanets, Analysis of Migration Paths in Fast-Ion Conductors with Voronoi–Dirichlet Partition. *Acta Crystallogr. B* **2006**, *62*, 1010.

[8] V. A. Blatov, Topological Relations between Three-Dimensional Periodic Nets. I. Uninodal Nets. *Acta Crystallogr A* **2007**, *63*, 329.

[9] S. P. Ong, W. D. Richards, A. Jain, G. Hautier, M. Kocher, S. Cholia, D. Gunter, V. L. Chevrier, K. A. Persson, G. Ceder, Python Materials Genomics (Pymatgen): A Robust, Open-Source Python Library for Materials Analysis. *Comput. Mater. Sci.* **2013**, *68*, 314.

[10] H. Pan, A. M. Ganose, M. Horton, M. Aykol, K. A. Persson, N. E. R. Zimmermann, A. Jain, Benchmarking Coordination Number Prediction Algorithms on Inorganic Crystal Structures. *Inorg. Chem.* **2021**, *60*, 1590.

[11] G. Venturini, R. Welter, B. Malaman, Crystallographic Data and Magnetic Properties of RT_6Ge_6 Compounds ($R \equiv$ Sc, Y, Nd, Sm, Gd–Lu; $T \equiv$ Mn, Fe). *J. Alloys Compd.* **1992**, *185*, 99.

[12] B. C. El Idrissi, G. Venturini, B. Malaman, Crystal Structures of RFe_6Sn_6 (R = Sc, Y, Gd–Tm, Lu) Rare-Earth Iron Stannides. *Mater. Res. Bull.* **1991**, *26*, 1331.

[13] P. Demchenko, O. Bodak, Rietveld Refinement of the Structure of $Nd_2Zn_{15}Ge_2$. *Pol. J. Chem.* **2001**, *75*, 153.

[14] J. B. Friauf, The Crystal Structure of Magnesium Di-Zincide. *Phys. Rev.* **1927**, *29*, 34.

[15] A. W. Hull, A New Method of X-Ray Crystal Analysis. *Phys. Rev.* **1917**, *10*, 661.

[16] K. Yoshihara, J. B. Taylor, L. D. Calvert, J. G. Despault, Rare-Earth Bismuthides. *J. Common Met.* **1975**, *41*, 329.

[17] M. A. Fremy, D. Gignoux, J. M. Moreau, D. Paccard, L. Paccard, RM_3Ga_2 Compounds ($R \equiv$ Rare Earth, $M \equiv$ Co, Ni): A New Structural Series. *J. Common Met.* **1985**, *106*, 251.

[18] V. Meschke, P. Gorai, V. Stevanović, E. S. Toberer, Search and Structural Featurization of Magnetically Frustrated Kagome Lattices. *Chem. Mater.* **2021**, *33*, 4373.

[19] N. Regnault, Y. Xu, M.-R. Li, D.-S. Ma, M. Jovanovic, A. Yazdani, S. S. P. Parkin, C. Felser, L. M. Schoop, N. P. Ong, R. J. Cava, L. Elcoro, Z.-D. Song, B. A. Bernevig, Catalogue of Flat-Band Stoichiometric Materials. *Nature* **2022**, *603*, 824.

[20] C. S. Chiu, A. N. Carroll, N. Regnault, A. A. Houck, Line-graph-lattice Crystal Structures of Stoichiometric Materials *Phys. Rev. Res.* **2022**, *4*, 023063

[21] M. Jovanovic, L. M. Schoop, Simple Chemical Rules for Predicting Band Structures of Kagome Materials. *J. Am. Chem. Soc.* **2022**, *144*, 10978.

[22] M. G. Vergniory, L. Elcoro, C. Felser, N. Regnault, B. A. Bernevig, Z. Wang, A Complete Catalogue of High-Quality Topological Materials. *Nature* **2019**, *566*, 480.

[23] M. I. Aroyo, J. M. Perez-Mato, D. Orobengoa, E. Tasci, G. De La Flor, A. Kirov, Crystallography Online: Bilbao Crystallographic Server. *Bulg. Chem. Commun.* **2011**, *43*, 183.

[24] M. G. Vergniory, L. Elcoro, C. Felser, N. Regnault, B. A. Bernevig, Z. Wang, A Complete Catalogue of High-Quality Topological Materials. *Nature* **2019**, *566*, 480.

[25] Y. Quan, Z. P. Yin, W. E. Pickett, Single Nodal Loop of Accidental Degeneracies in Minimal Symmetry: Triclinic $CaAs_3$. *Phys. Rev. Lett.* **2017**, *118*, 176402

[26] P. Villars, K. Cenzual, Pearson's Crystal Data: Crystal Structure Database for Inorganic Compounds (Release 2023/24), **2023**.

[27] Y. V. Verbovytskyy, I. Y. Zavaliy, V. V. Berezovets, P. Y. Lyutyy, Solid Gas and Electrochemical Hydrogenation Properties of the $R_{1-x}R'_xMgNi_{4-y}Co_y$ (R, R' = Y, La, Ce) Alloys. *Phys. Chem. Solid State* **2020**, *21*, 503.

[28] R. K. Jain, A. Jain, I. P. Jain, Effect of La-Content on the Hydrogenation Properties of the $Ce_{1-x}La_xNi_3Cr_2$ (x=0.2, 0.4, 0.6, 0.8, 1) Alloys. *Int. J. Hydrog. Energy* **2012**, *37*, 3683.

[29] Y. Zhang, Y.-C. Luo, D.-H. Wang, F.-L. Zhang, J.-C. Kang, R.-X. Yan, Investigation on Crystal Structure and Electrochemical Properties of $LaMgNi_{3.7}M_{0.3}$ (M=Ni, Al, Mn, Co, Sn, Cu) Hydrogen Storage Alloys. *Gongneng Cailiao/Journal Funct. Mater.* **2005**, *36*, 1372.

[30] Y. Verbovytskyy, Y. Kosarchyn, I. Zavaliy, Solid Gas and Electrochemical Hydrogenation Properties of the Selected R,$R'MgNi_{4-x}M_x$ (R,R' = La, Pr, Nd; M = Fe, Mn; x = 0.5, 1) Alloys. *Fr.-Ukr. J. Chem.* **2020**, *8*, 126.

[31] J.-L. Bobet, B. Chevalier, B. Darriet, Crystallographic and Hydrogen Sorption Properties of $TiMn_2$ Based Alloys. *Intermetallics* **2000**, *8*, 359.

[32] J.-L. Bobet, B. Darriet, Relationship between Hydrogen Sorption Properties and Crystallography for $TiMn_2$ Based Alloys. *Int. J. Hydrog. Energy* **2000**, *25*, 767.

[33] M. Yoshida, E. Akiba, Hydrogen Absorbing-Desorbing Properties and Crystal Structure of the Zr–Ti–Ni–Mn–V AB_2 Laves Phase Alloys. *J. Alloys Compd.* **1995**, *224*, 121.

[34] V. O. Oprysk, Yu. V. Verbovytskyy, V. V. Shtender, P. Ya. Lyutyy, I. Yu. Zavaliy, The $Pr_{1-x}La_xMgNi_{4-y}Co_y$ Alloys: Synthesis, Structure and Hydrogenation Properties. *Solid State Sci.* **2018**, *84*, 112.

[35] J.-H. Lee, K.-Y. Lee, S.-M. Lee, J.-Y. Lee, Self-Discharge Characteristics of Sealed Ni–MH Batteries Using $Zr_{1-x}Ti_xV_{0.8}Ni_{1.6}$ Anodes. *J. Alloys Compd.* **1995**, *221*, 174.

[36] A. Züttel, F. Meli, L. Schlapbach, Surface and Bulk Properties of the $Ti_yZr_{1-y}(V_xNi_{1-x})_2$ Alloy System as Active Electrode Material in Alkaline Electrolyte. *J. Alloys Compd.* **1995**, *231*, 645.

[37] D.-Y. Yan, G. Sandrock, S. Suda, Zr–Ti–V–Ni Alloys with Dendrite-Free Structure. *J. Alloys Compd.* **1995**, *223*, 32.

[38] H. Shi, S. Han, Y. Jia, Y. Liu, X. Zhao, B. Liu, Investigations on Hydrogen Storage Properties of $LaMg_{8.52}Ni_{2.23}M_{0.15}$ (M=Ni, Cu, Cr) Alloys. *J. Rare Earths* **2013**, *31*, 79.

[39] D.-M. Kim, S.-M. Lee, K.-J. Jang, J.-Y. Lee, The Electrode Characteristics of Over-Stoichiometric $ZrMn_{0.5}V_{0.5}Ni_{1.4+y}$ (y=0.0, 0.2, 0.4 and 0.6) Alloys with C15 Laves Phase Structure. *J. Alloys Compd.* **1998**, *268*, 241.

[40] G. Venturini, Structural and Magnetic Properties of $REMn_6Sn_{\approx 4}Ge_{\approx 2}$ Compounds (RE = Sc, Y, Nd, Sm, Gd–Tm, Lu). *J. Alloys Compd.* **2005**, *398*, 42.

[41] C. Lefèvre, G. Venturini, B. Malaman, Neutron Diffraction Study of the Magnetocrystalline Anisotropy in $TbMn_6Sn_{5.8}Ga_{0.2}$, $TbMn_6Sn_5Ga$, $HoMn_6Sn_5Ga$ and $HoMn_6Sn_5In$ Compounds. *J. Alloys Compd.* **2003**, *358*, 29.

[42] D. Lupu, A. R. Biriş, E. Indrea, A. S. Biriş, G. Bele, L. Schlapbach, A. Züttel, Hydrogen Absorption and Hydride Electrode Behaviour of the Laves Phase $ZrV_{1.5-x}Cr_xNi_{1.5}$. *J. Alloys Compd.* **1999**, *291*, 289.

[43] M. Bououdina, H. Enoki, E. Akiba, The Investigation of the $Zr_{1-y}Ti_y(Cr_{1-x}Ni_x)_2$–$H_2$ System $0.0 \leqslant y \leqslant 1.0$ and $0.0 \leqslant x \leqslant 1.0$ Phase Composition Analysis and Thermodynamic Properties. *J. Alloys Compd.* **1998**, *281*, 290.

[44] G. Kresse, J. Furthmüller, Efficient Iterative Schemes for Ab Initio Total-Energy Calculations Using a Plane-Wave Basis Set. *Phys. Rev. B* **1996**, *54*, 11169.

[45] J. P. Perdew, K. Burke, M. Ernzerhof, Generalized Gradient Approximation Made Simple. *Phys Rev Lett* **1996**, *77*, 3865.

[46] S. L. Dudarev, G. A. Botton, S. Y. Savrasov, C. J. Humphreys, A. P. Sutton, Electron-Energy-Loss Spectra and the Structural Stability of Nickel Oxide: An LSDA+U Study. *Phys. Rev. B* **1998**, *57*, 1505.

[47] P. Söderlind, P. E. A. Turchi, A. Landa, V. Lordi, Ground-State Properties of Rare-Earth Metals: An Evaluation of Density-Functional Theory. *J. Phys. Condens. Matter* **2014**, *26*, 416001.

[48] J. Jensen, A. R. Mackintosh, *Rare Earth Magnetism: Structures and Excitations*., Oxford University PressOxford **1991**.

[49] G. Dhakal, F. Cheenicode Kabeer, A. K. Pathak, F. Kabir, N. Poudel, R. Filippone, J. Casey, A. Pradhan Sakhya, S. Regmi, C. Sims, K. Dimitri, P. Manfrinetti, K. Gofryk, P. M. Oppeneer, M. Neupane, Anisotropically Large Anomalous and Topological Hall Effect in a Kagome Magnet. *Phys. Rev. B* **2021**, *104*, L161115.
